# Emergence of Irregular Activity in Networks of Strongly Coupled Conductance-Based Neurons

**DOI:** 10.1103/physrevx.12.011044

**Published:** 2022-03-08

**Authors:** A. Sanzeni, M. H. Histed, N. Brunel

**Affiliations:** 1 Center for Theoretical Neuroscience, Columbia University, New York, New York, USA; 2 Department of Neurobiology, Duke University, Durham, North Carolina, USA; 3 National Institute of Mental Health Intramural Program, NIH, Bethesda, Maryland, USA; 4 Department of Physics, Duke University, Durham, North Carolina, USA

**Keywords:** Biological Physics, Complex Systems, Interdisciplinary Physics

## Abstract

Cortical neurons are characterized by irregular firing and a broad distribution of rates. The balanced state model explains these observations with a cancellation of mean excitatory and inhibitory currents, which makes fluctuations drive firing. In networks of neurons with current-based synapses, the balanced state emerges dynamically if coupling is strong, i.e., if the mean number of synapses per neuron *K* is large and synaptic efficacy is of the order of $1/\sqrt{K}$. When synapses are conductance-based, current fluctuations are suppressed when coupling is strong, questioning the applicability of the balanced state idea to biological neural networks. We analyze networks of strongly coupled conductance-based neurons and show that asynchronous irregular activity and broad distributions of rates emerge if synaptic efficacy is of the order of 1/ log(*K*). In such networks, unlike in the standard balanced state model, current fluctuations are small and firing is maintained by a drift-diffusion balance. This balance emerges dynamically, without fine-tuning, if inputs are smaller than a critical value, which depends on synaptic time constants and coupling strength, and is significantly more robust to connection heterogeneities than the classical balanced state model. Our analysis makes experimentally testable predictions of how the network response properties should evolve as input increases.

## I. INTRODUCTION

Each neuron in the cortex receives inputs from hundreds to thousands of presynaptic neurons. If these inputs were to sum to produce a large net current, the central limit theorem argues that fluctuations should be small compared to the mean, leading to regular firing, as observed during *in vitro* experiments under constant current injection [1,2]. Cortical activity, however, is highly irregular, with a coefficient of variation of interspike intervals (CV of ISI) close to one [3,4]. To explain the observed irregularity, it has been proposed that neural networks operate in a balanced state, where strong feed forward and recurrent excitatory inputs are canceled by recurrent inhibition and firing is driven by fluctuations [5,6]. At the single-neuron level, in order for this state to emerge, input currents must satisfy two constraints. First, excitatory and inhibitory currents must be fine-tuned to produce an average input below threshold. Specifically, if *K* and *J* represent the average number of input connections per neuron and synaptic efficacy, respectively, the difference between excitatory and inhibitory presynaptic inputs must be of the order of 1/*KJ*. Second, input fluctuations should be large enough to drive firing.

It has been shown that the balanced state emerges dynamically (without fine-tuning) in randomly connected networks of binary units [7,8] and networks of current-based spiking neurons [9,10], provided that coupling is strong, and recurrent inhibition is powerful enough to counterbalance instabilities due to recurrent excitation. However, these results are all derived assuming that the firing of a presynaptic neuron produces a fixed amount of synaptic current, hence neglecting the dependence of synaptic current on the membrane potential, a key aspect of neuronal biophysics. In real synapses, synaptic inputs are mediated by changes in conductance, due to opening of synaptic receptor channels on the membrane, and synaptic currents are proportional to the product of synaptic conductance and a driving force which depends on the membrane potential. Models that incorporate this description are referred to as “conductance-based synapses”.

Large synaptic conductances have been shown to have major effects on the stationary [11] and dynamical [12] response of single cells and form the basis of the “high-conductance state” [13–19] that has been argued to describe well *in vivo* data [20–22] (but see Ref. [23] and Sec. IX). At the network level, conductance modulation plays a role in controlling signal propagation [24], input summation [25], interactions between traveling waves [26], and firing statistics [27]. However, most of the previously mentioned studies rely exclusively on numerical simulations, and, in spite of a few attempts at analytical descriptions of networks of conductance-based neurons [17,28–32], an understanding of the behavior of such networks when coupling is strong is still lacking.

Here, we investigate networks of strongly coupled conductance-based neurons. We find that, for synapses of the order of $1/\sqrt{K}$, fluctuations are too weak to sustain firing, questioning the relevance of the balanced state idea to cortical dynamics. Our analysis, on the other hand, shows that stronger synapses [of the order of 1/log (*K*)] generate irregular firing when coupling is strong. We characterize the properties of networks with such a scaling, showing that they match properties observed in the cortex, and discuss constraints induced by the synaptic time constants. The model generates qualitatively different predictions compared to the current-based model, which could be tested experimentally.

## II. MODELS OF SINGLE-NEURON AND NETWORK DYNAMICS

### A. Membrane potential dynamics

We study the dynamics of networks of leaky integrate-and-fire (LIF) neurons with conductance-based synaptic inputs. The membrane potential *V*_*j*_ of the *j*th neuron in the network follows the equation

(1) $$C_{j}\frac{dV_{j}}{dt}=-\underset{A=L,E,I}{\sum }g_{A}^{j}\left(V_{j}-E_{A}\right),$$

where $C_{j}$ is the neuronal capacitance; *E*_*L*_, *E*_*E*_, and *E*_*I*_ are the reversal potentials of the leak, excitatory, and inhibitory currents, respectively; while $g_{L}^{j}$, $g_{E}^{j}$, and $g_{I}^{j}$ are the leak, excitatory, and inhibitory conductances, respectively. Assuming instantaneous synapses (the case of finite synaptic time constants is discussed in Sec. VIII), excitatory and inhibitory conductances are given by

(2) $$\frac{g_{E,I}^{j}}{g_{L}^{j}}=\tau_{j}\underset{m}{\sum }a_{jm}\underset{n}{\sum }\delta \left(t-t_{m}^{n}\right).$$

In Eq. (2), $\tau_{j}=C_{j}/g_{L}^{j}$ is the single-neuron membrane time constant, *a*_*jm*_ are dimensionless measures of synaptic strength between neuron *j* and neuron *m*, and $\sum_{n}\delta \left(t-t_{m}^{n}\right)$ represents the sum of all the spikes generated at times $t_{m}^{n}$ by neuron *m*. Every time the membrane potential *V*_*j*_ reaches the firing threshold *θ*, the *j*th neuron emits a spike, and its membrane potential is set to a reset *V*_*r*_ and stays at that value for a refractory period *τ*_*rp*_; after this time, the dynamics resumes, following Eq. (1).

We use *a*_*jm*_ = *a* (*ag*) for all excitatory (inhibitory) synapses. In the homogeneous case, each neuron receives synaptic inputs from *K*_*E*_ = *K* (*K*_*I*_ = *γK*) excitatory (inhibitory) cells. In the network case, each neuron receives additional *K*_*X*_ = *K* excitatory inputs from an external population firing with Poisson statistics with rate *ν*_*X*_. We use excitatory and inhibitory neurons with the same biophysical properties; hence, the above assumptions imply that the firing rates of excitatory and inhibitory neurons are equal; *ν* = *ν*_*E*_ = *ν*_*I*_. Models taking into account the biophysical diversity between the excitatory and inhibitory populations are discussed in the Appendix D. When heterogeneity is taken into account, the above-defined values of *K*_*E*,*I*,*X*_ represent the means of Gaussian distributions. We use the following single-neuron parameters: *τ*_*rp*_ = 2 ms, *θ* = −55 mV, *V*_*r*_ = −65 mV, *E*_*E*_ = 0 mV, *E*_*I*_ −75 mV, *E*_*L*_ = −80 mV, and *τ*_*j*_ = *τ*_*L*_ = 20 ms. We explore various scalings of *a* with *K*, and, in all cases, we assume that *a* ≪ 1. When *a* ≪ 1, an incoming spike produced by an excitatory presynaptic neuron produces a jump in the membrane potential of amplitude *a*(*E*_*E*_ – *V*), where *V* is the voltage just before spike arrival. In the cortex, *V* ~ −60 mV and average amplitudes of postsynaptic potentials are on the order of 0.5–1.0 mV [33–39]. Thus, we expect realistic values of *a* to be on the order of 0.01.

### B. Diffusion and effective time constant approximations

We assume that each cell receives projections from a large number of cells (*K* ≫ 1), neurons are sparsely connected and fire approximately as Poisson processes, each incoming spike provides a small change in conductance (*a* ≪ 1), and temporal correlations in synaptic inputs can be neglected. Under these assumptions, we can use the diffusion approximation and approximate the conductances as

(3) $$\frac{g_{E}}{g_{L}}=a\tau_{L}\left[Kr_{E}+\sqrt{Kr_{E}}\zeta_{E}\right],\;\frac{g_{I}}{g_{L}}=ag\tau_{L}\left[\gamma Kr_{I}+\sqrt{\gamma Kr_{I}}\zeta_{I}\right],$$

where *r*_*E*_ and *r*_*I*_ are the firing rates of presynaptic *E* and *I* neurons, respectively, and *ζ*_*E*_ and *ζ*_*I*_ are independent Gaussian white noise terms with zero mean and unit variance density. In the single-neuron case, we take *r*_*E*_ = *ν*_*X*_, *r*_*I*_ = *ην*_*X*_, where *η* represents the ratio of *I*/*E* input rate. In the network case, *r*_*E*_ = *ν*_*X*_ + *ν*, *r*_*I*_ = *ν*, where *ν*_*X*_ is the external rate, while *ν* is the firing rate of excitatory and inhibitory neurons in the network, determined self-consistently (see below). We point out that, for some activity levels, the assumption of Poisson presynaptic firing made in the derivation of Eq. (3) breaks down, as neurons in the network show interspike intervals with CV significantly different from one [e.g., see Fig. 3(c)]. However, comparisons between mean field results and numerical simulations (see Appendix E) show that neglecting non-Poissonianity [as well as other contributions discussed above Eq. (3)] generates quantitative but not qualitative discrepancies, with magnitude that decreases with coupling strength. Moreover, in Appendix B, we show that if *a* ≪ 1, the firing of neurons in the network matches that of a Poisson process with a refractory period and, hence, when *ν* ≪ 1/*τ*_*rp*_, deviations from Poissonianity become negligible.

Using the diffusion approximation, Eq. (1) reduces to

(4) $$\tau \frac{dV}{dt}=-V+\mu +\sigma (V)\sqrt{\tau }\zeta ,$$

where *ζ* is a white noise term, with zero mean and unit variance density, while

(5) $$\tau^{-1}=\tau_{L}^{-1}+aK\left(r_{E}+r_{I}g\gamma \right),\;\mu =\tau \left\{E_{L}/\tau_{L}+aK\left[r_{E}E_{E}+r_{I}g\gamma E_{I}\right]\right\},\;\sigma^{2}(V)=a^{2}K\tau \left[r_{E}{\left(V-E_{E}\right)}^{2}+g^{2}\gamma r_{I}{\left(V-E_{I}\right)}^{2}\right].$$

In Eq. (4), *τ* is an effective membrane time constant, while *μ* and *σ*^2^(*V*) represent the average and the variance of the synaptic current generated by incoming spikes, respectively.

The noise term in Eq. (4) can be decomposed into an additive and a multiplicative component. The latter has an effect on membrane voltage statistics that is of the same order of the contribution coming from synaptic shot noise [40], a factor which is neglected in deriving Eq. (3). Therefore, for a consistent analysis, we neglect the multiplicative component of the noise in the above derivation; this leads to an equation of the form of Eq. (4) with the substitution

(6) σ(V)→σ(μ).

This approach is termed the effective time constant approximation [40]. Note that the substitution of Eq. (6) greatly simplifies mathematical expressions, but it is not a necessary ingredient for the results presented in this paper. In fact, all our results can be obtained without having to resort to this approximation (see Appendixes A, B, and D).

### C. Current-based model

The previous definitions and results translate directly to current-based models, with the only exception that the dependency of excitatory and inhibitory synaptic currents on the membrane potential are neglected (see Ref. [10] for more details). Therefore, Eq. (1) becomes

(7) $$\tau_{j}\frac{dV_{j}}{dt}=-V_{j}+I_{E}^{j}-I_{I}^{j},$$

where

$$I_{A}^{j}=\tau_{j}\sum_{m}J_{jm}\sum_{n}\delta \left(t-t_{m}^{n}\right)$$

represent the excitatory and inhibitory input currents. Starting from Eq. (7), making assumptions analogous to those discussed above and using the diffusion approximation [10], the dynamics of current-based neurons is given by an equation of the form of Eq. (4) with

(8) $$\tau =\tau_{L},\;\mu =\tau JK\left[r_{E}-g\gamma r_{I}\right],\;\sigma^{2}=\tau J^{2}K\left[r_{E}+g^{2}\gamma r_{I}\right].$$

Note that, unlike what happens in conductance-based models, *τ* is a fixed parameter and does not depend on the network firing rate or external drive. Another difference between the current-based and conductance-based models is that in the latter, but not the former, model *σ* depends on *V*; as we discuss above, this difference is neglected in the main text, where we use the effective time constant approximation.

## III. BEHAVIOR OF SINGLE-NEURON RESPONSE FOR LARGE *K*

We start our analysis by investigating the effects of synaptic conductance on single-neuron response. We consider a neuron receiving *K* (*γK*) excitatory (inhibitory) inputs, each with synaptic efficacy *J* (*gJ*), from cells firing with Poisson statistics with a rate

(9) $$r_{E}=\nu_{X},\;r_{I}=\eta \nu_{X}$$

and analyze its membrane potential dynamics in the frameworks of current-based and conductance-based models. In both models, the membrane potential *V* follows a stochastic differential equation of the form of Eq. (4); differences emerge in the dependency of *τ*, *μ*, and *σ* on the parameters characterizing the connectivity, *K* and *J*. In particular, in the current-based model, the different terms in Eq. (8) can be written as

$$\tau \sim \tau_{0}^{\text{curr }},\;\mu \sim KJ\mu_{0}^{\text{curr }},\;\sigma \sim \sqrt{K}J\sigma_{0}^{\text{curr }},$$

where $\tau_{0}^{\text{curr }}$, $\mu_{0}^{\text{curr }}$, and $\sigma_{0}^{\text{curr }}$ are independent of *J* and *K*. In the conductance-based model, the efficacy of excitatory and inhibitory synapses depend on the membrane potential as *J* = *a*(*E*_*E*,*I*_ − *V*); the different terms in Eq. (4), under the assumption that *Ka* ≫ 1, become of the order of

$$\tau \sim \frac{\tau_{0}^{\text{cond }}}{Ka},\;\mu \sim \mu_{0}^{\text{cond }},\;\sigma \sim \sqrt{a}\sigma_{0}^{\text{cond }}.$$

Here, all these terms depend on parameters in a completely different way than in the current-based case. As we show below, these differences drastically modify how the neural response changes as *K* and *J* are varied and, hence, the size of *J* ensuring a finite response for a given value of *K*.

**FIG. 1.**
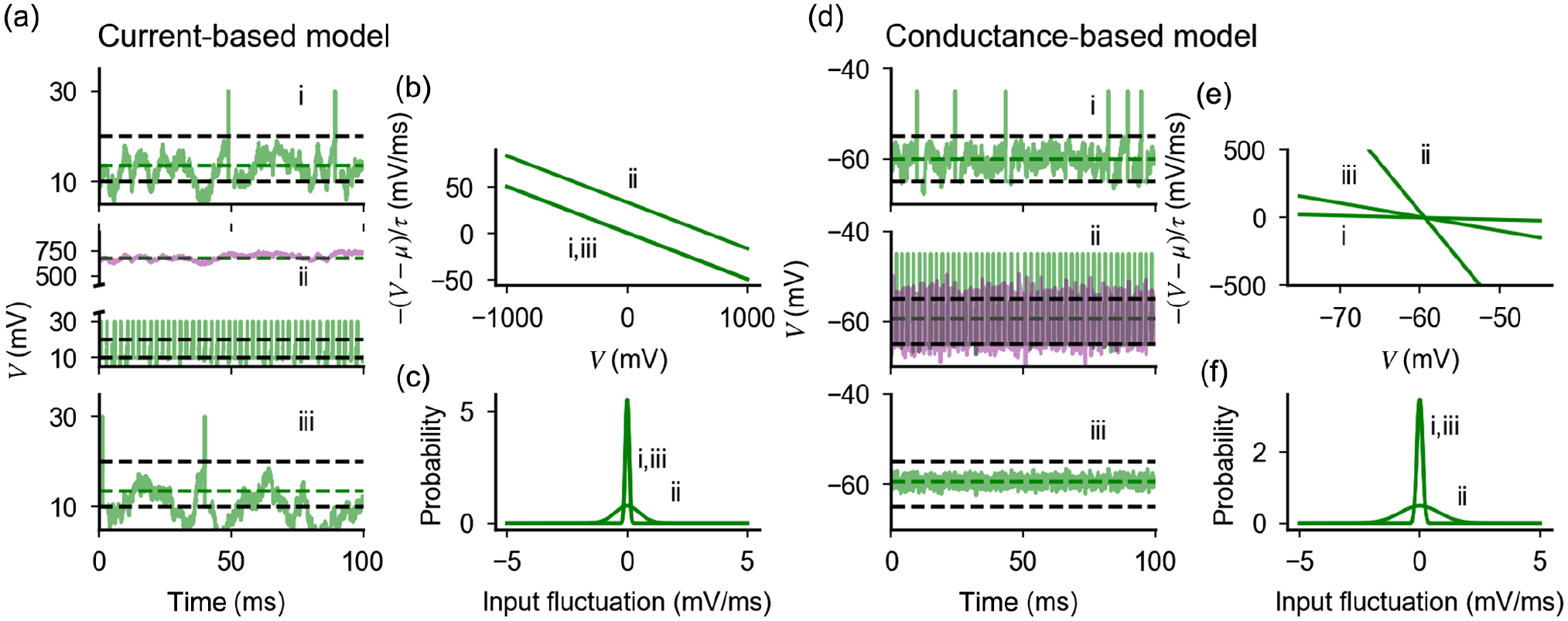
Effects of coupling strength on the firing behavior of current-based and conductance-based neurons. (a) Membrane potential of a single current-based neuron for (i) *J* = 0.3 mV, *K* = 10^3^, *g* = *γ* = 1, and *η* such that 1 − *gγη* = 0.075; (ii) with *K* = 5 × 10^4^; (iii) with *K* = 5 × 10^4^ and scaled synaptic efficacy ($J\sim 1/\sqrt{K}$, which gives *J* = 0.04 mV) and input difference 1 − *gγη* = 0.01; (b),(c) Effect of coupling strength on drift force and input noise in a current-based neuron. (d) Membrane potential of a single conductance-based neuron for fixed input difference (*g*1 − *γη* = −2.8) and (i) *a* = 0.01, *K* = 10^3^; (ii) *K* = 5 × 10^4^; (iii) *K* = 5 × 10^4^ and scaled synaptic efficacy ($a\sim 1/\sqrt{K}$, *a* = 0.001). (e),(f) Effect of coupling strength on drift force and input noise in a conductance-based neuron. In (a) and (d), dashed lines represent the threshold and reset (black) and equilibrium value of membrane potential (green). In (a) (ii) and (d)(ii), light purple traces represent dynamics in absence of a spiking mechanism. Input fluctuations in (c) and (f) represent input noise per unit time, i.e., the integral of $\sigma \sqrt{\tau }\zeta$ of Eq. (4) computed over an interval Δ*t* and normalized over Δ*t*.

The dynamics of a current-based neuron is shown in Fig. 1(a)(i), with parameters leading to irregular firing. Because of the chosen parameter values, the mean excitatory and inhibitory inputs approximately cancel each other, generating subthreshold average input and fluctuation-driven spikes, which leads to irregularity of firing. If all parameters are fixed while *K* is increased (*J* ~ *K*^0^), the response changes drastically [Fig. 1(a)(ii)], since the mean input becomes much larger than threshold and firing becomes regular. To understand this effect, we analyze how terms in Eq. (4) are modified as *K* increases. The evolution of the membrane potential in time is determined by two terms: a drift term −(*V* − *μ*)/*τ*, which drives the membrane potential toward its mean value *μ*, and a noise term $\sigma /\sqrt{\tau }$, which leads to fluctuations around this mean value. Increasing *K* modifies the equilibrium value *μ* of the drift force and the input noise, which increase proportionally to *KJ*(1 − *γgη*) and *KJ*^2^(*γg*^2^*η +* 1), respectively [Figs. 1(b) and 1(c)]. This observation)suggests that, to preserve irregular firing as *K* is increased, two ingredients are needed. First, the rates of excitatory and inhibitory inputs must be fine-tuned to maintain a mean input below threshold; this can be achieved by choosing *γgη* − 1 ~ 1/*KJ*. Second, the amplitude of input fluctuations should be preserved; this can be achieved by scaling synaptic efficacy as $J\sim 1/\sqrt{K}$. Once these two conditions are met, irregular firing is restored [Fig. 1(a)(iii)]. Importantly, in a network with $J\sim 1/\sqrt{K}$, irregular firing emerges without fine-tuning, since rates dynamically adjust to balance excitatory and inhibitory inputs and maintain mean inputs below threshold [7,8].

We now show that the above solution does not work once synaptic conductance is taken into account. The dynamics of a conductance-based neuron in response to the inputs described above is shown in Fig. 1(d)(i). As in the current-based neuron, it features irregular firing, with mean input below threshold and spiking driven by fluctuations, and firing becomes regular for larger *K*, leaving all other parameters unchanged [Fig. 1(d)(ii)]. However, unlike the current-based neuron, input remains below threshold at large *K*; regular firing is produced by large fluctuations, which saturate the response and produce spikes that are regularly spaced because of the refractory period. These observations can be understood by inspecting the equation for the membrane potential dynamics [Eq. (4)]: increasing *K* leaves invariant the equilibrium value of the membrane potential *μ* but increases the drift force and the input noise amplitude as *Ka* and $\sqrt{K}a$, respectively [Figs. 1(e) and 1(f)]. Since the equilibrium membrane potential is fixed below threshold, response properties are determined by the interplay between drift force and input noise, which have opposite effects on the probability of spike generation. The response saturation observed in Fig. 1(d)(ii) shows that, as *K* increases at fixed *a*, fluctuations dominate over drift force. On the other hand, using the scaling $a\sim 1/\sqrt{K}$ leaves the amplitude of fluctuations unchanged but generates a restoring force of the order of $\sqrt{K}$ [Fig. 1(e)] which dominates and completely abolishes firing at strong coupling [Fig. 1(d)(iii)].

Results in Fig. 1 show that the response of a conductance-based neuron when *K* is large depends on the balance between drift force and input noise. The scalings *a* ~ *O*(1) and $a\sim 1/\sqrt{K}$ leave one of the two contributions dominant, suggesting that an intermediate scaling could keep a balance between them. Below, we derive such a scaling, showing that it preserves firing rate and CV of ISI when *K* becomes large.

## IV. A SCALING RELATION THAT PRESERVES SINGLE-NEURON RESPONSE FOR LARGE *K*

We analyze under what conditions the response of a single conductance-based neuron is preserved when *K* is large. For a LIF neuron described by Eqs. (4)–(6), the single cell transfer function, i.e., the dependency of the firing rate *ν* on the external drive *ν*_*X*_, is given by [41,42]

(10) $$\nu ={\left[\tau_{rp}+\tau \sqrt{\pi }\int_{v_{\text{min}}}^{v_{\text{max}}}dx\text{ exp}\left(x^{2}\right)[1+\text{erf}(x)]\right]}^{-1},$$

with

(11) $$v(x)=\frac{x-\mu }{\sigma },\;v_{\text{min}}=v\left(V_{r}\right),\;v_{\text{max}}=v(\theta ).$$

In the biologically relevant case of *a* ≪ 1, Eq. (10) simplifies significantly, using the fact that *v*_max_, the distance between the average membrane potential and the threshold, is of the order of $1/\sqrt{a}$. Therefore, *v*_max_ is large when *a* is small; in this limit, the firing rate is given by the Kramers escape rate [43], and Eq. (10) becomes

(12) $$\nu =\frac{1}{\tau_{rp}+\frac{Q}{\nu_{X}}},\;Q=\frac{\bar{\tau }\sqrt{\pi }}{\sqrt{a}K\bar{v}}\text{exp}\left(\frac{{\bar{v}}^{2}}{a}\right),$$

where we define ${\bar{v}}^{2}=av_{\text{max}}^{2}$ and $\bar{\tau }=aK\nu_{X}\tau$. The motivation to introduce $\bar{v}$ and $\bar{\tau }$ is that they remain of the order of 1 in the small *a* limit, provided the external inputs *ν*_*X*_ are at least of the order of 1/(*aKτ*_*L*_). When the external inputs are such that *ν*_*X*_ ≫ 1/(*aKτ*_*L*_), these quantities become independent of *ν*_*X*_, and *a* and *K* are given by

(13) $$\bar{\tau }={\left(1+g\gamma \eta \right)}^{-1},\;\bar{v}=\frac{\theta -\bar{\mu }}{\bar{\sigma }},\;\bar{\mu }=\bar{\tau }\left(E_{E}+g\gamma \eta E_{I}\right),\;{\bar{\sigma }}^{2}=\bar{\tau }\left[{\left(\bar{\mu }-E_{E}\right)}^{2}+g^{2}\gamma \eta {\left(\bar{\mu }-E_{I}\right)}^{2}\right].$$

The firing rate given by Eq. (12) remains finite when *a* is small and/or *K* is large if Q remains of the order of one; this condition leads to the following scaling relationship:

(14) $$K\sim \frac{\bar{\tau }}{\sqrt{a}\bar{v}}\text{exp}\left(\frac{{\bar{v}}^{2}}{a}\right);$$

i.e., *a* should be of the order of 1/log *K*.

In Appendix C, we show that expressions analogous to Eq. (12) can be derived in integrate-and-fire neuron models which feature additional intrinsic voltage-dependent currents, as long as synapses are conductance based and input noise is small (*a* ≪ 1). Examples of such models include the exponential integrate-and-fire neurons with its spike-generating exponential current [44] and models with voltage-gated subthreshold currents [23]. Moreover, we show that, in these models, firing remains finite if *a* ~ 1/ log(*K*), and voltage-dependent currents generate corrections to the logarithmic scaling which are negligible when coupling is strong.

**FIG. 2.**
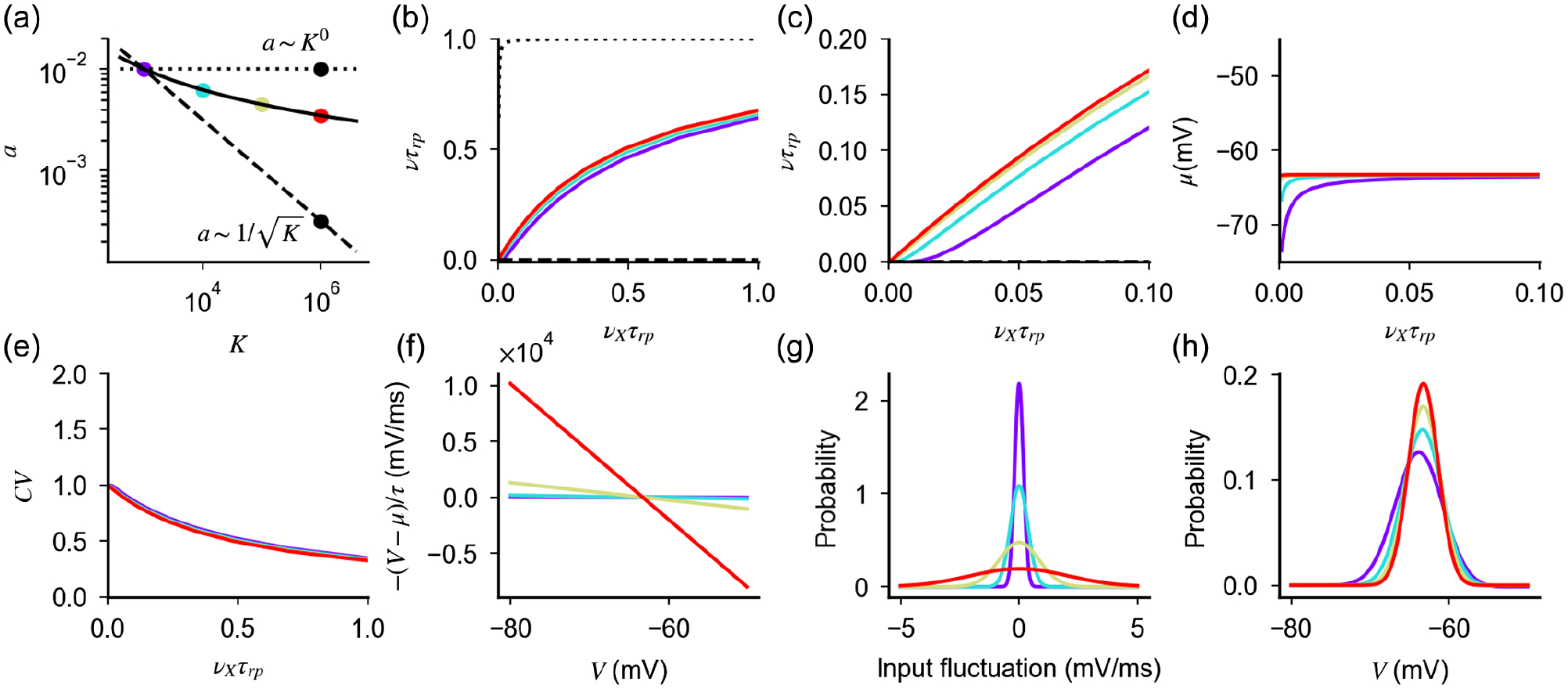
The scaling of Eq. (14) preserves the response of a single conductance-based neuron for large *K*. (a) The scaling relation preserving firing in conductance-based neurons [Eq. (14), solid line]; constant scaling (*a* ~ *K*^0^, dotted line) and scaling of the balanced state model ($a\sim 1/\sqrt{K}$, dashed line) are shown as a comparison. Colored dots indicate values of *a* and *K* used subsequently. (b)–(h) Response of conductance-based neurons, for different values of the coupling strength and synaptic efficacy (colored lines). The scaling of Eq. (14) preserves how the firing rate (b),(c), equilibrium value of the membrane potential (d), and CV of the interspike interval distribution (e) depend on external input rate *ν*_*X*_. This invariance is achieved by increasing the drift force (f) and input fluctuation (g) in a way that weakly decreases (logarithmically in *K*) membrane potential fluctuations (h). Different scalings either saturate or suppress the response [(b); black lines correspond to *K* = 10^5^ and *a* values as in (a)]. Parameters: *a* = 0.01 for *K* = 10^3^, *g* = 12, *η* = 1.8, and *γ* = 1/4.

In Fig. 2(a), we compare the scaling defined by Eq. (14) with the $a\sim 1/\sqrt{K}$ scaling of current-based neurons. At low values of *K*, the values of *a* obtained with the two scalings are similar; at larger values of *K*, synaptic strength defined by Eq. (14) decays as *a* ~ 1/log(*K*)—i.e., synapses are stronger in the conductance-based model than in the current-based model. Examples of single-neuron transfer function computed from Eq. (10) for different coupling strength are shown in Figs. 2(b) and 2(c). Responses are nonlinear at onset and close to saturation. As predicted by the theory, scaling *a* with *K* according to Eq. (14) preserves the firing rate over a region of inputs that increases with the coupling strength [Figs. 2(c) and 2(d)], while the average membrane potential remains below threshold [Fig. 2(d)]. The quantity $\bar{v}/\sqrt{a}$ represents the distance from threshold of the equilibrium membrane potential in units of input fluctuations; Eq. (14) implies that this distance increases with the coupling strength. When *K* is very large, the effective membrane time constant, which is of the order of *τ* ~ 1/*aKν*_*X*_, becomes small and firing is driven by fluctuations that, on the timescale of this effective membrane time constant, are rare.

We next investigate if the above scaling preserves irregular firing by analyzing the CV of interspike intervals. This quantity is given by [10]

(15) $${\text{CV}}^{2}=2\pi \nu^{2}\tau^{2}\int_{v_{\text{min}}}^{v_{\text{max}}}dxe^{x^{2}}\int_{-\infty }^{x}dye^{y^{2}}{\left[1+\text{erf}(y)\right]}^{2}$$

and, for the biologically relevant case of *a* ≪ 1 and *μ* < *θ*, reduces to (see Appendix B for details)

(16) $$\text{CV}=1-\tau_{rp}\nu ;$$

i.e., the CV is close to one at low rates, and it decays monotonically as the neuron approaches saturation. Critically, Eq. (16) depends on the coupling strength only through *ν*; hence, any scaling relation preserving firing rate also produces a CV of the order of one at a low rate. We validate numerically this result in Fig. 2(e).

We now investigate how Eq. (14) preserves irregular firing in conductance-based neurons. We have shown that increasing *K* at fixed *a* produces large input and membrane fluctuations, which saturate firing; the scaling $a\sim 1/\sqrt{K}$ preserves input fluctuations but, because of the strong drift force, suppresses membrane potential fluctuations and, hence, firing. The scaling of Eq. (14), at every value of *K*, yields the value of *a* that balances the contribution of drift and input fluctuations, so that membrane fluctuations are of the right size to preserve the rate of threshold crossing. Note that, unlike what happens in the current-based model, both input fluctuations and drift force increase with *K* [Figs. 2(f) and 2(g)], while the membrane potential distribution, which is given by [45]

(17) $$P(V)=\frac{2\nu \tau }{\sigma }\int_{v(V)}^{v_{\text{max}}}dx\theta \left[x-v\left(V_{r}\right)\right]\text{exp}\left[x^{2}-v{\left(V\right)}^{2}\right],$$

slowly becomes narrower [Fig. 2(h)]. This result can be understood by noticing that, when *a* ≪ 1 and neglecting the contribution due to the refractory period, Eq. (17) reduces to

(18) $$P(V)=\frac{1}{\sigma \sqrt{\pi }}\text{exp}\left(-\frac{{\left(V-\mu \right)}^{2}}{\sigma^{2}}\right).$$

Hence, the probability distribution becomes Gaussian when coupling is strong, with a variance proportional to *σ*^2^ ~ *a*. We note that, since *a* is of the order of 1/ log *K*, the width of the distribution becomes small only for unrealistically large values of *K*.

## V. ASYNCHRONOUS IRREGULAR ACTIVITY IN NETWORK RESPONSE AT STRONG COUPLING

We have so far considered the case of a single neuron subjected to stochastic inputs. We now show how the above results generalize to the network case, where inputs to a neuron are produced by a combination of external and recurrent inputs.

We consider networks of recurrently connected excitatory and inhibitory neurons, firing at rate *ν*, stimulated by an external population firing with Poisson statistics with firing rate *ν*_*X*_. Using again the diffusion approximation, the response of a single neuron in the networks is given by Eq. (10) [and, hence, Eq. (12)] with

(19) $$r_{E}=\nu_{X}+\nu ,\;r_{I}=\nu .$$

**FIG. 3.**
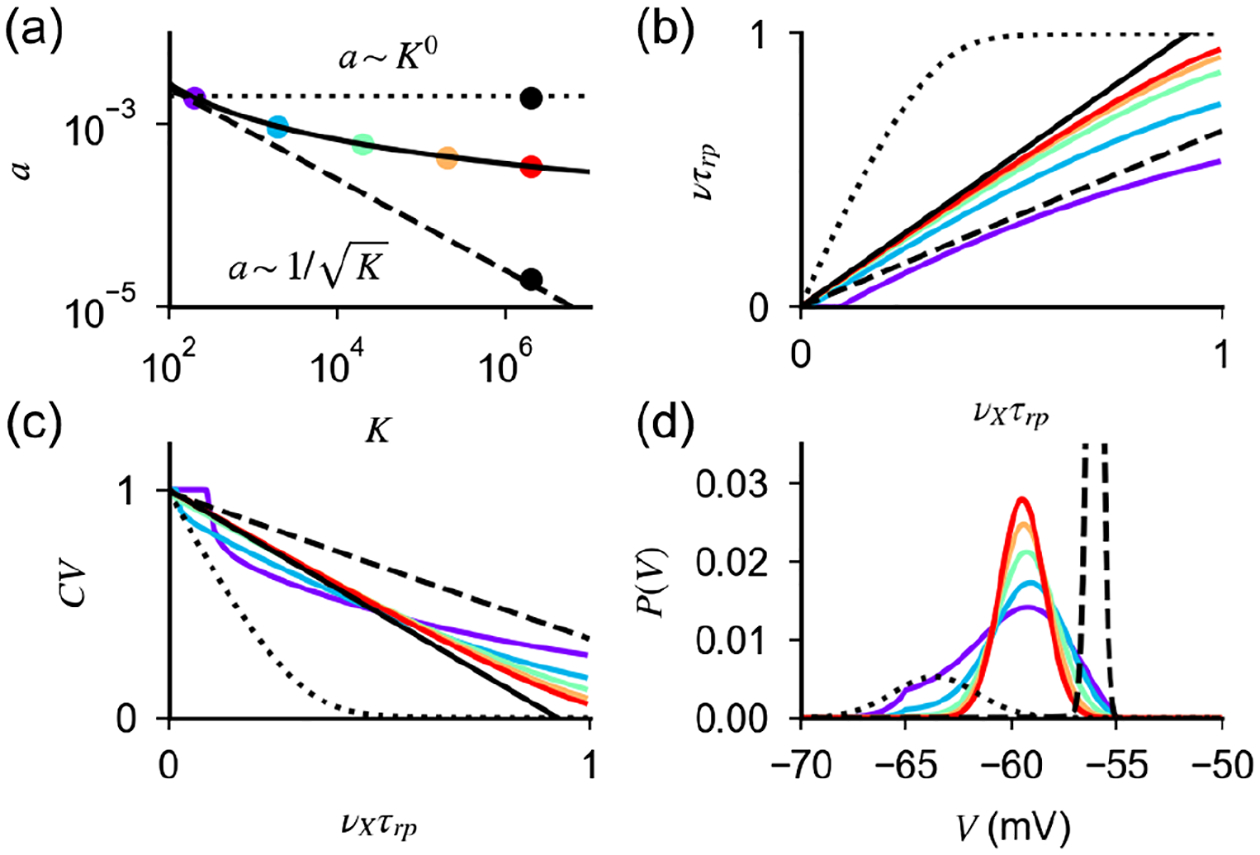
Response of networks of conductance-based neurons for large *K*. (a) Scaling relation defined by self-consistency condition given by Eqs. (14) and (19) (black line), values of parameters used in (b)–(d) (colored dots). Constant scaling (*a* ~ *K*^0^ dotted line) and scaling of the balanced state model ($a\sim 1/\sqrt{K}$, dashed line) are shown for comparison. (b),(c) Firing rate and CV of ISI as a function of the external input, obtained from Eqs. (10) and (15) (colored lines) with the strong-coupling limit solution of Eqs. (20) and (16) (black line). (d) Probability distribution of the membrane potential obtained from Eq, (17). In (b)–(d), dotted and dashed lines represent quantities obtained with the scalings *J* ~ *K*^0^ and $J\sim 1/\sqrt{K}$, respectively, for values of *K* and *J* indicated in (a) (black dots). Parameters: *γ* = 1/4 and *g* = 30.

Equation (10), if all neurons in a given population are described by the same single-cell parameters and the network is in an asynchronous state in which cells fire at a constant firing rate, provides an implicit equation whose solution is the network transfer function. Example solutions are shown in Fig. 3(b) (numerical validation of the mean field results is provided in Appendix E). In Appendix D, we prove that firing in the network is preserved when coupling is strong if parameters are rescaled according to Eq. (14). Moreover, we show that response nonlinearities are suppressed and the network response in the strong-coupling limit (i.e., when *K* goes infinity) is given, up to saturation, by

(20) $$\nu =\rho \nu_{X}.$$

The parameter *ρ*, which is obtained by solving Eq. (12) self-consistently (see Appendix D for details), is the response gain in the strong-coupling limit. Finally, our derivation implies that Eq. (14) preserves irregular firing and creates a probability distribution of membrane potential whose width decreases only logarithmically as *K* increases [Figs. 3(c) and 3(d) and numerical validation in Appendix E], as in the single-neuron case. While this logarithmic decrease is a qualitative difference with the current-based balanced state in which the width stays finite in the large *K* limit, in practice, for realistic values of *K*, realistic fluctuations of membrane potential (a few mV) can be observed in both cases.

We now turn to the question of what happens in networks with different scalings between *a* and *K*. Our analysis of single-neuron response described above shows that scalings different from that of Eq. (14) fail to preserve firing for large *K*, as they let either input noise or drift dominate. However, the situation in networks might be different, since recurrent interactions could, in principle, adjust the statistics of input currents such that irregular firing at low rates is preserved when coupling becomes strong. Thus, we turn to the analysis of the network behavior when a scaling *a* ~ *K*^−*α*^ is assumed. For *α* ≤ 0, the dominant contribution of input noise at the single-neuron level (Figs. 1 and 2) generates saturation of response and regular firing in the network (Fig. 3). This can be understood by noticing that, for large *K*, the factor Q in Eq. (12) becomes negligible and the self-consistency condition defining the network rate is solved by *ν* = 1/*τ*_*rp*_. For *α* > 0, the network response for large *K* is determined by two competing elements. On the one hand, input drift dominates and tends to suppress firing (Figs. 1 and 2). On the other hand, for the network to be stable, inhibition must dominate recurrent interactions [9]. Hence, any suppression in network activity reduces recurrent inhibition and tends to increase neural activity. When these two elements conspire to generate a finite network response, the factor Q in Eq. (12) must be of the order of one and $\bar{v}\sim a\sim K^{-\alpha }$. In this scenario, the network activity exhibits the following features (Fig. 3): (i) the mean inputs drive neurons very close to threshold $\left(\theta -\bar{\mu }\sim a\bar{\sigma }\sim K^{-\alpha }\right)$; (ii) the response of the network to external inputs is linear and, up to corrections of the order of *K*^−*α*^, given by

(21) $$\nu =\frac{\left(E_{E}-\theta \right)\nu_{X}}{\theta (1+g\gamma )-E_{E}-g\gamma E_{I}};$$

(iii) firing is irregular [because of Eq. (16)]; (iv) the width of the membrane potential distribution is of the order of *a* ~ *K*^−*α*^ [because of Eq. (18)]. Therefore, scalings different from that in Eq. (14) can produce asynchronous irregular activity in networks of conductance-based neurons, but this leads to networks with membrane potentials narrowly distributed close to threshold, a property which seems at odds with what is observed in the cortex [46–51].

## VI. ROBUST LOG-NORMAL DISTRIBUTION OF FIRING RATES IN NETWORKS WITH HETEROGENEOUS CONNECTIVITY

Up to this point, we have assumed a number of connections equal for all neurons. In real networks, however, this number fluctuates from cell to cell. The goal of this section is to analyze the effects of heterogeneous connectivity in networks of conductance-based neurons.

**FIG. 4.**
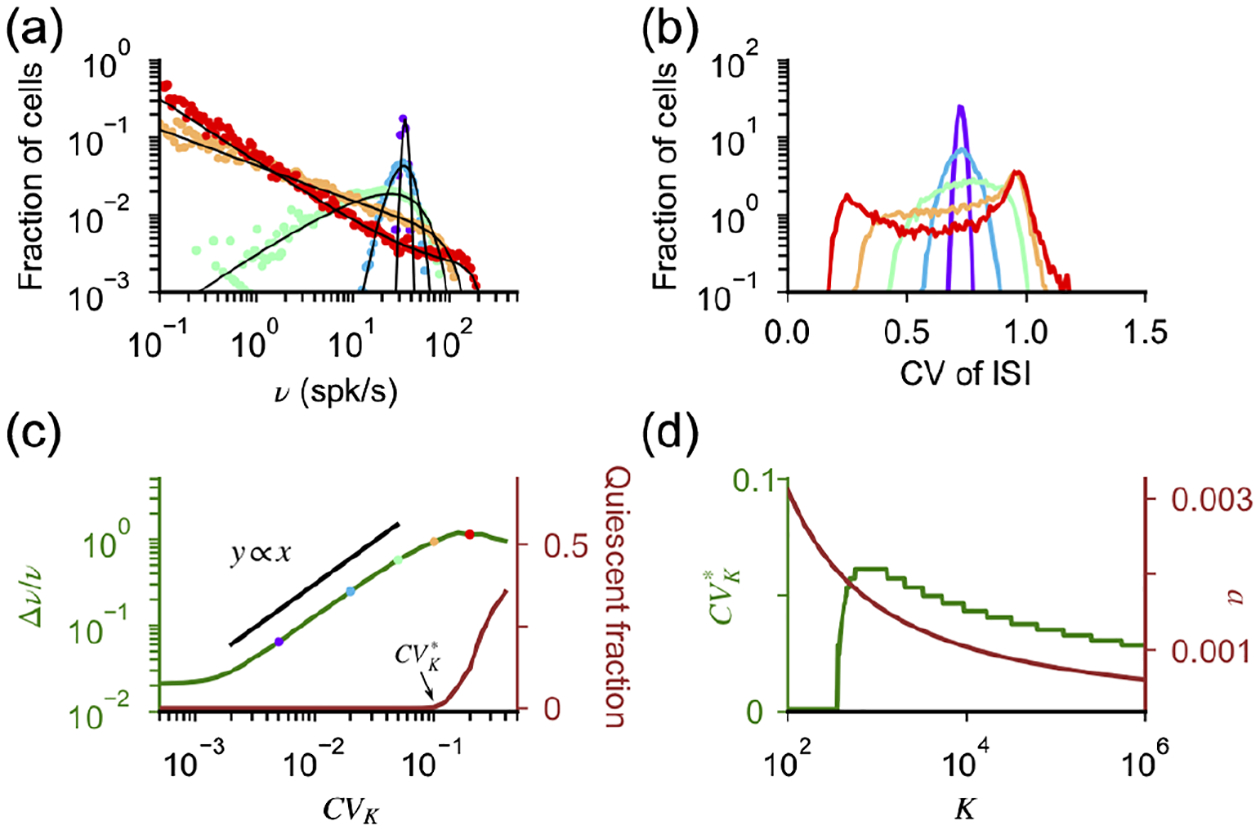
Effects of heterogeneous connectivity on the network response. (a),(b) Distribution of *ν* and CV of ISI computed from network simulations (dots) and from the mean field analysis [(a), black lines] for different values of CV_*K*_ [values are indicated by dots in (c)]. (c) Δ*ν*/*ν* (green, left axis) l Communication within Local and fraction of quiescent cells (brown, right axis) computed from network simulations as a function of CV_*K*_. For ${\text{CV}}_{K}≲{\text{CV}}_{K}^{*},\Delta \nu /\nu$, Δ*ν*/*ν* increases linearly, as predicted by the mean field analysis; deviations from linear scaling emerge for ${\text{CV}}_{K}≳{\text{CV}}_{K}^{*}$, when a significant fraction of cells become quiescent. The deviation from linear scaling at low CV_*K*_ is due to a sampling error in estimating the firing rate from simulations. (d) ${\text{CV}}_{K}^{*}$ as a function of *K* computed from the mean field theory (green, left axis), with *a* rescaled according to Eq. (14). For large *K*, ${\text{CV}}_{K}^{*}$ decays proportionally to *a* (brown, right axis). When *K* is too low, the network is silent and ${\text{CV}}_{K}^{*}=0$. In (a)–(c), *K* = 10^3^, *g* = 20, *a* = 1.6 × 10^−3^, *N*_*E*_ = *N*_*X*_ = *N*_*I*_/*γ* = 10*K*, and *ν*_*X*_ = 0.05/*τ*_*rp*_. In network simulations, the dynamics is run for 20 s using a time step of 50 *μ*s. Parameters in (d) are as in Fig. 3.

We investigate numerically the effects of connection heterogeneity as follows. We choose a Gaussian distribution of the number of connections per neuron, with mean *K* and variance Δ*K*^2^ for excitatory connections and mean *γK* and variance *γ*^2^Δ*K*^2^ for inhibitory connections. The connectivity matrix is constructed by drawing first randomly *E* and *I* in-degrees $K_{E,X,I}^{i}$ from these Gaussian distributions for each neuron and then selecting at random $K_{E,X,I}^{i}$ *E*/*I* presynaptic neurons. We then simulate network dynamics and measure the distribution of rates and CV of the ISI in the population. Results for different values of CV_*K*_ Δ*K*/*K* are shown in Figs. 4(a)–4(c). For small and moderate values of connection heterogeneity, increasing CV_*K*_ broadens the distribution of rates and CV of the ISI, but both distributions remain peaked around a mean rate that is close to that of homogeneous networks [Figs. 4(a) and 4(b)]. For larger CV_*K*_, on the other hand, the distribution of rates changes its shape, with a large fraction of neurons moving to very low rates, while others increase their rates [Fig. 4(a)] and the distribution of the CV of ISI becomes bimodal, with a peak at low CV corresponding to the high-rate neurons, while the peak at a CV close to 1 corresponds to neurons with very low firing rates [Fig. 4(b)].

To characterize more systematically the change in the distribution of rates with CV_*K*_, we measure, for each value of CV_*K*_, the fraction of quiescent cells, defined as the number of cells that do not spike during 20 s of the simulated dynamics [Fig. 4(c)]. This analysis shows that the number of quiescent cells, and, hence, the distribution of rates, changes abruptly as the CV_*K*_ is above a critical value ${\text{CV}}_{K}^{*}$. Importantly, unlike our definition of the fraction of quiescent cells, this abrupt change is a property of the network that is independent of the duration of the simulation.

To understand these numerical results, we perform a mean field analysis of the effects of connection heterogeneity on the distribution of rates (Appendix F). This analysis captures quantitatively numerical simulations [Fig. 4(a)] and shows that, in the limit of small CV_*K*_ and *a*, rates in the network are given by

(22) $$\nu_{i}=\nu_{0}\text{exp}\left[\Omega \frac{{\text{CV}}_{K}}{a}z_{i}\right],$$

where *ν*_0_ is the population average in the absence of heterogeneity, *z*_*i*_ is a Gaussian random variable, and the prefactor Ω is independent of *a*, *K*, and *ν*_*X*_. The exponent in Eq. (22) represents a quenched disorder in the value of *v*^*i*^, i.e., in the distance from threshold of the single cell *μ*^*i*^ in units of input noise. As shown in Appendix F, Eq. (22) implies that the distribution of rates is log-normal, a feature consistent with experimental observations [52–54] and distributions of rates in networks of current-based LIF neurons [55]. It also implies that the variance of the distribution Δ*ν*/*ν* should increase linearly with CV_*K*_, a prediction which is confirmed by numerical simulations [Fig. 4(c)]. The derivation in Appendix F also provides an explanation for the change in the shape of the distribution for larger CV_*K*_. In fact, for larger heterogeneity, the small CV_*K*_ approximation is not valid, and fluctuations in input connectivity produce cells for which *μ*^*i*^ far from *θ*, that are firing either at an extremely low rate (*μ*^*i*^ < *θ*) or regularly (*μ*^*i*^ > *θ*). The latter generates the peak at low values in the CV of the ISI seen for large values CV_*K*_.

The quantity ${\text{CV}}_{K}^{*}$ represents the level of connection heterogeneity above which significant deviations from the asynchronous irregular state emerges; i.e., large fractions of neurons show extremely low or regular firing. Equation (22) suggests that ${\text{CV}}_{K}^{*}$ should increase linearly with *a*. We validate this prediction with our mean field model, by computing the minimal value of CV_*K*_ at which 1% of the cells fire at a rate of 10^−3^ spk/s [Fig. 4(d)]. Note that the derivation of Eq. (22) assumes only *a* to be small and does not depend on the scaling relation between *a* and *K*. On the other hand, the fact that ${\text{CV}}_{K}^{*}$ increases linearly with *a* makes the state emerging in networks of conductance-based neurons with *a* ~ 1/ log(*K*) significantly more robust to connection fluctuations than that emerging with *a* ~ *K*^−*α*^, for which ${\text{CV}}_{K}^{*}\sim K^{-\alpha }$, and with current-based neurons, where ${\text{CV}}_{K}^{*}\sim 1/\sqrt{K}$ [56]. Note that, while in randomly connected networks ${\text{CV}}_{K}\sim 1/\sqrt{K}$, a larger degree of heterogeneity is observed in cortical networks [50,56–62]. Our results show that networks of conductance-based neurons could potentially be much more robust to such heterogeneities than networks of current-based neurons.

## VII. COMPARISON WITH EXPERIMENTAL DATA

**FIG. 5.**
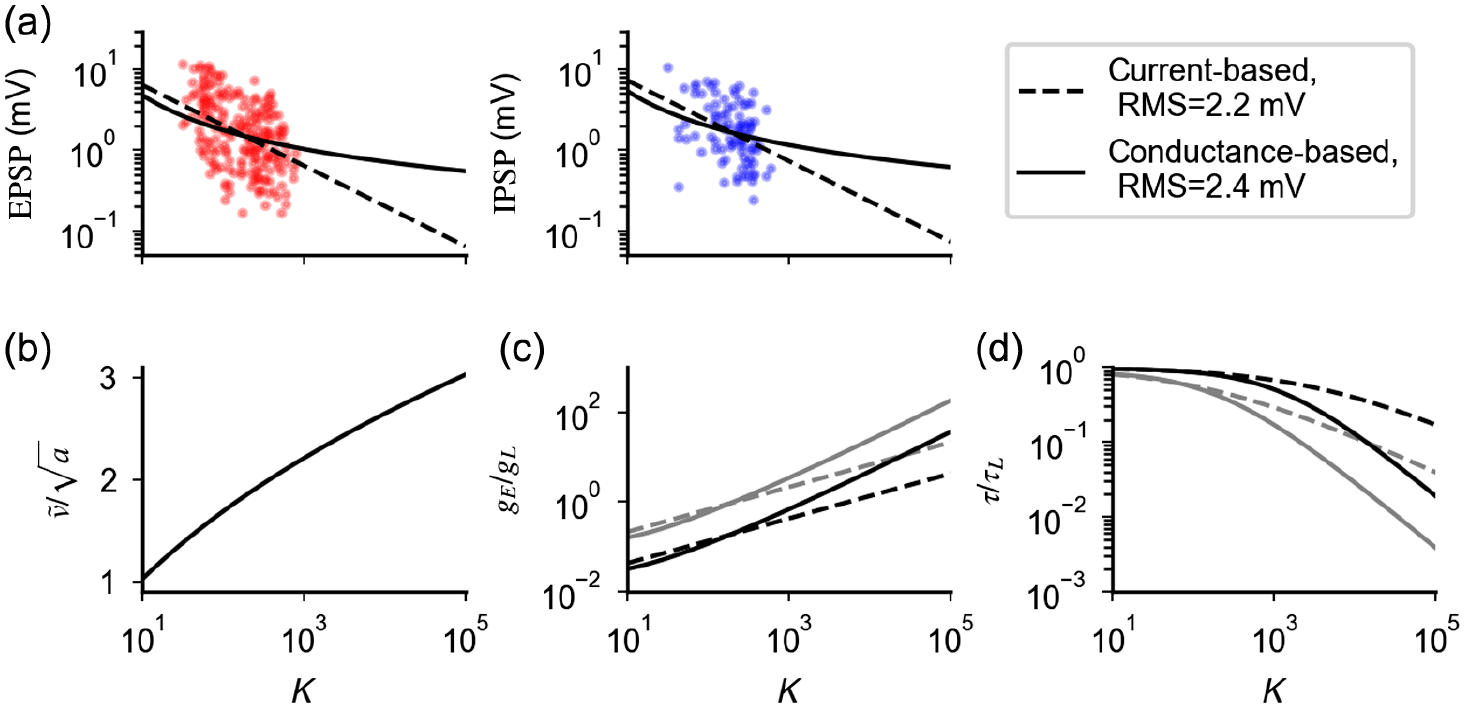
Comparison of predictions given by current-based and the conductance-based models in describing experimental data from cultures. (a) Strength of excitatory (EPSP) and inhibitory (IPSP) postsynaptic potentials recorded in Ref. [63] are compared with best fits using scaling relationships derived from networks with current-based synapses (dashed line) and conductance-based synapses (continuous line). Root mean square (rms) and best fit parameters are rms = 2.2 mV, *g* = 1.1, and *J*_0_ = 20 mV for the current-based model and rms = 2.4 mV, *g* = 3.4, and $\bar{v}=0.08$ for the conductance-based model. (b) Value of $\bar{v}/\sqrt{a}$ predicted by the conductance-based model as a function of *K*. (c) Ratio between excitatory and leak conductance as a function of *K*, for *ν*_*E*_ = *ν*_*I*_ = *ν*_*X*_ = 1 spk/s (black) and *ν*_*E*_ = *ν*_*I*_ = *ν*_*X*_ = 5 spk/s (gray) obtained with *a* rescaled as Eq. (14) (continuous line) and as $1/\sqrt{K}$ (dashed line). (d) Ratio between *τ* and *τ*_*L*_ as a function of *K*; parameters and scaling as in (c).

The relation between synaptic efficacy and number of connections per neuron has been recently studied experimentally using a culture preparation [63]. In this paper, it is found that cultures in which *K* is larger have weaker synapses than cultures with smaller *K* (Fig. 5). In what follows, we compare these data with the scalings expected in networks of current-based and conductance-based neurons and discuss implications for *in vivo* networks.

In the current-based model, the strength of excitatory and inhibitory postsynaptic potentials as a function of *K* can be written as $J_{E}=J_{0}/\sqrt{K}$ and *J*_*I*_ = *gJ*_*E*_, respectively. In the conductance-based model, these quantities become *J*_*E*_ = (*V* − *E*_*E*_)*a* and *J*_*I*_ = *g*(*V* − *E*_*I*_)*a*, where $a=a(K,\bar{v})$ is given by Eq. (14) while, for the dataset of Ref. [63], *V* ~ −60 mV, *J*_*E*_ ~ *J*_*I*_, *E*_*E*_ ~ 0 mV, and *E*_*I*_ ~ −80 mV. For each model, we infer free parameters from the data with a least-squares optimization in logarithmic scale (best fit, *g* = 1.1 and *J*_0_ = 20 mV in the current-based model and *g* = 3.4 and $\bar{v}=0.08$ in the conductance-based model) and compute the expected synaptic strength as a function of *K* [lines in Fig. 5(a)]. Our analysis shows that the performances of the current-based and the conductance-based model in describing the data, over the range of *K* explored in the experiment, are similar, with the former being slightly better than the latter (root mean square 2.2 vs 2.4 mV). This result is consistent with the observation made in Ref. [63] that, when fitted with a power law *J* ~ *K*^−*β*^, data are best described by *β* = 0.59 but are compatible with a broad range of values (95% confidence interval [0.47:0.70]). Note that, even though both models give similar results for PSP amplitudes in the range of values of *K* present in cultures (approximately 50–10 00), they give significantly different predictions for larger values of *K*. For instance, for *K* = 10 000, *J*_*E*_ is expected to be approximately 0.2 mV in the current-based model and approximately 0.7 mV in the conductance-based model.

In Fig. 5(b), we plot the distance between the equilibrium membrane potential *μ* and threshold *θ* in units of input fluctuations and $\bar{v}/\sqrt{a}$ as a function of *K* using the value of $\bar{v}$ obtained above and find that the expected value *in vivo*, where *K* ~ 10^3^–10^4^, is in the range 2–3. In Figs. 5(c) and 5(d), we plot how total synaptic excitatory conductance and the effective membrane time constant change as a function of *K*. Both quantities change significantly faster using the conductance-based scaling [*g*_*E*_/*g*_*L*_ ~ *K*/ log (*K*); *τ*/*τ*_*L*_ ~ log (*K*)/*K*] than expected by the scaling of the current-based model $\left(g_{E}/g_{L}\sim \sqrt{K};\tau /\tau_{L}\sim 1/\sqrt{K}\right)$. For *K* in the range 10^3^–10^4^ and mean firing rates in the range 1–5 spk/s, the total synaptic conductance is found be in a range from about 2 to 50 times the leak conductance, while the effective membrane time constant is found to be smaller than the membrane time constant by a factor of 2–50. We compare these values with available experimental data in Sec. IX.

## VIII. EFFECTS OF FINITE SYNAPTIC TIME CONSTANTS

Results discussed in previous sections show that the effective membrane time constant *τ* decreases with presynaptic activity and with coupling strength. This observation raises the question whether the assumption of negligible synaptic time constants we have made in our analysis is reasonable. Synaptic decay time constants of experimentally recorded postsynaptic currents range from a few milliseconds (for AMPA and GABA_*A*_ receptor-mediated currents) to tens of milliseconds (for GABA_*B*_ and NMDA receptor-mediated currents; see, e.g., Ref. [64]); i.e., they are comparable to the membrane time constant already at weak coupling, where *τ* ~ *τ*_*L*_ is typically in the range 10–30 ms [65]. Interestingly, experiments suggest that synaptic dynamics might be faster in physiological conditions (e.g., Ref. [66] finds a 0.5 ms decay time constant for the AMPA receptor at 35°C). Nonetheless, in the strong-coupling limit, the effective membrane time constant goes to zero, and so our assumption of negligible synaptic time constant clearly breaks down in that limit. In this section, we analyze models with finite coupling strength and show that synaptic dynamics modifies the drift-diffusion balance characteristic of conductance-based models, making it input dependent. At the end of the section, we discuss how this input-dependent drift-diffusion balance can be preserved in the strong-coupling limit.

**FIG. 6.**
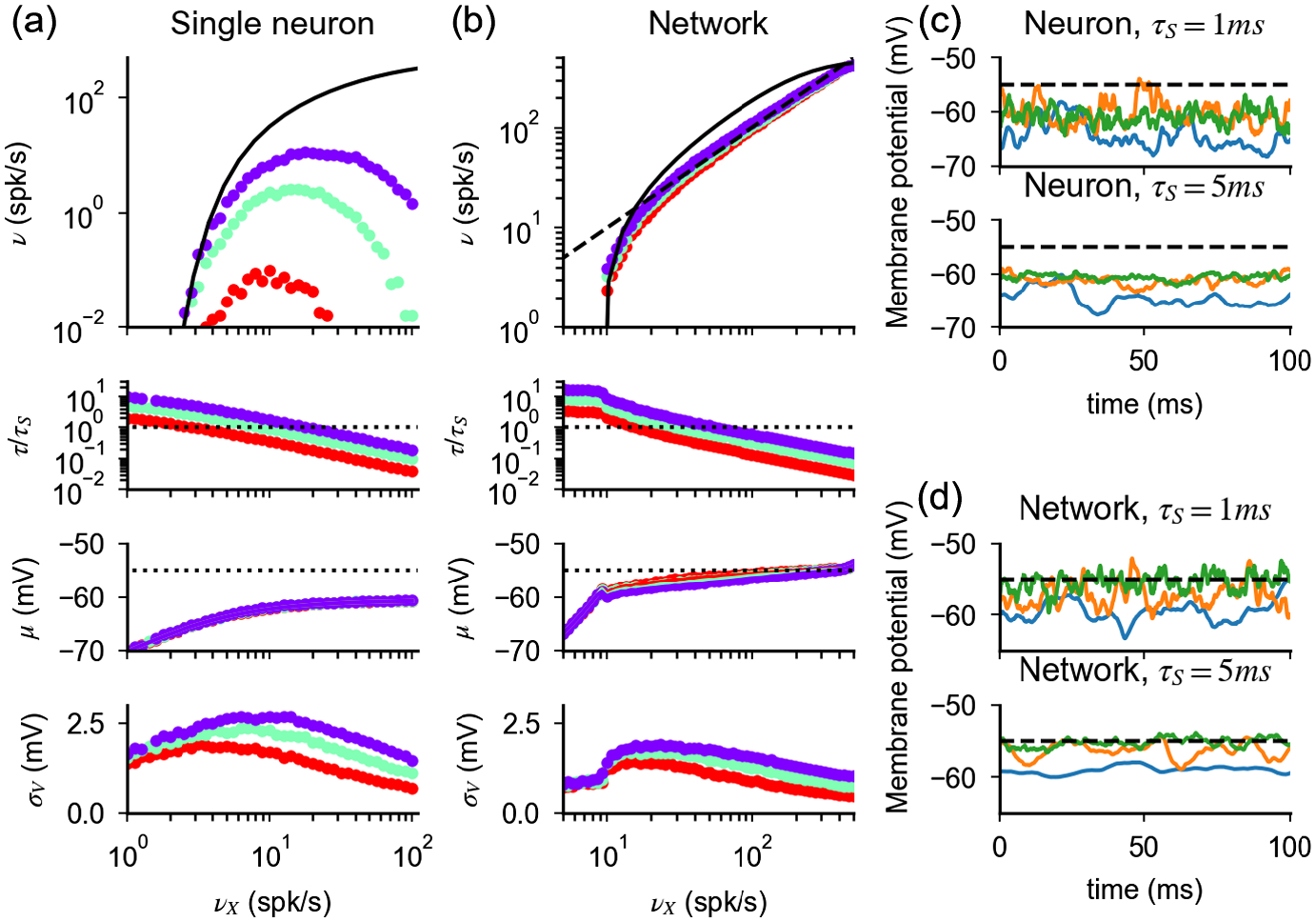
Effects of synaptic time constant on single-neuron and network response. (a) Single-neuron response as a function of input rate *ν*_*X*_, computed numerically from Eqs. (1) and (23). Different colors correspond to different values of *τ*_*S*_ (purple, 1 ms; blue, 2 ms; red, 5 ms). Firing rates (first row) match predictions obtained for instantaneous synapses (lines) for small *τ*_*S*_/*τ*; significant deviations and response suppression emerge for larger *τ*_*S*_/*τ*. The effective membrane time constant (*τ*, second row) decreases with the input rate and reaches the value *τ*_*S*_/*τ* ~ 1 (dashed line) for lower levels of external drive when *τ*_*S*_ is larger. The equilibrium value of the membrane potential (*μ*, third row) increases with the input rate and is independent of *τ*_*S*_ (black dotted line represents the spiking threshold). The magnitude of fluctuations of the membrane potential (*σ*_*V*_, fourth row) has a nonmonotonic relationship with the input rate and peaks at a value of *ν*_*X*_ for which *τ* is of the same order as *τ*_*S*_. (b) Analogous to (a) but in the network case. Firing rates are no longer suppressed as *τ*_*S*_/*τ* increases but approach the response scaling predicted by Eq. (21) (dashed line). As discussed in the text, high firing rates are obtained by increasing the value of *μ* toward threshold. (c) Examples of membrane potential dynamics for a single neuron in the absence of spiking mechanisms and for two different values of *τ*_*S*_. Colors correspond to increasing *ν*_*X*_ = 5 (blue), 40 (orange), and 100 spk/s (green), respectively. High-frequency fluctuations are suppressed as *ν*_*X*_ increases. (d) Analogous to (c) but in the network case and for *ν*_*X*_ = 5, 40, and 100 spk/s. Increasing *ν*_*X*_ reduces recurrent inhibition and produces membrane potential trajectories which are increasingly closer to the firing threshold. Simulations parameters are *K* = 10^3^, *a* = 0.01, *g* = 12, *η* = 1.4, and *γ* 1/4 (single neuron); *K* = 10^3^, *a* = 0.002, *g* = 22, and *γ* = 1/4 (network). Simulations are performed with the simulator brian2 [71], with neurons receiving inputs from independent Poisson units firing at rates *Kν*_*X*_ and *γKην*_*X*_, in the single-neuron case, or *Kν*_*X*_, in the network case. Network simulations use *N*_*E,I*_ = 10*K* excitatory and inhibitory neurons.

With finite synaptic time constants, the temporal evolution of conductances in Eq. (2) is replaced by

(23) $$\tau_{E,I}\frac{dg_{E,I}^{j}}{dt}=-g_{E,I}^{j}+g_{L}^{j}\tau_{E,I}\underset{m}{\sum }a_{jm}\underset{n}{\sum }\delta \left(t-t_{m}^{n}\right),$$

where *τ*_*E*_/*τ*_*I*_ are the decay time constant of *E*/*I* synaptic conductances, respectively. The single-neuron membrane potential dynamics is described by Eqs. (1) and (23). Here, for simplicity, we take excitatory and inhibitory synaptic currents to have the same decay time constant: *τ*_*E*_ = *τ*_*I*_ = *τ*_*S*_. Figure 6(a) shows how the synaptic time constant modifies the mean firing rate of single integrate-and-fire neurons in response to *K* (*γK*) excitatory (inhibitory) inputs with synaptic strength *a* (*ga*) and frequency *ν*_*X*_ (*ην*_*X*_). The figure shows that, though the mean firing rate is close to predictions obtained with instantaneous synapses for low *ν*_*X*_, deviations emerge as input increases and firing is strongly suppressed for large *ν*_*X*_. To understand these numerical results, we resort again to the diffusion approximation [67,68], together with the effective time constant approximation [11,69], to derive a simplified expression of the single-neuron membrane potential dynamics with finite synaptic time constant (details in Appendix G):

(24) $$\tau \frac{dV}{dt}=-(V-\mu )+\sigma \sqrt{\frac{\tau }{\tau_{S}}}z,$$

where *τ*, *μ*, and *σ* are as in the case of negligible synaptic time constant [Eq. (5)] while *z* is an Ornstein-Uhlenbeck process with correlation time *τ*_*S*_. Thus, compared to the instantaneous synapse case [Eq. (4)], input fluctuations with frequency larger than 1/*τ*_*S*_ are suppressed, and, for large *τ*_*S*_/*τ*, the membrane potential dynamics is given by

(25) $$V(t)=\mu +\sigma \sqrt{\frac{\tau }{\tau_{S}}}z(t);$$

i.e., the membrane potential is essentially slaved to a time-dependent effective reversal potential given by the rhs of Eq. (25) [14]. Note that Eq. (25) is valid only in the subthreshold regime. When the rhs of Eq. (25) exceeds the threshold, the neuron fires a burst of action potentials whose frequency, in the strong-coupling limit, is close to the inverse of the refractory period [70]. As *ν*_*X*_ increases, the equilibrium value *μ* remains constant while *τ* decreases, leading to a suppression of membrane fluctuations [Figs. 6(a) and 6(c)] and, in turn, to the suppression of response observed in Fig. 6(a). Therefore, the filtering of synaptic input induced by synaptic dynamics breaks the drift-diffusion balance which supports firing in conductance-based neurons. In Appendix H, we show that the suppression of the single-neuron firing rate described here cannot be prevented by short-term synaptic plasticity.

We next examine the effect of a finite synaptic time constant on network response. Numerically computed responses in networks of neurons with a finite synaptic time constant are shown in Fig. 6(b). The network response is close to the prediction obtained with instantaneous synapses for small *τ*_*S*_/*τ*, and deviations emerge for *τ*_*S*_/*τ* ~ 1. Hence, analogously to the single-neuron case, network properties discussed in the case of instantaneous synapses remain valid for low inputs. However, unlike the single-neuron case, no suppression appears for larger *τ*_*S*_/*τ*. This lack of suppression in the network response, analogously to the one we discuss in networks with instantaneous synapses and *a* ~ *K*^−*α*^, is a consequence of the fact that, to have stable dynamics when *K* is large, inhibition must dominate recurrent interactions [9]. In this regime, any change which would produce suppression of single-neuron response (e.g., increase of *ν*_*X*_) lowers recurrent inhibition and increases the equilibrium value of the membrane potential *μ* [Figs. 6(b) and 6(d)]. The balance between these two effects determines the network firing rate and, when *τ*_*S*_/*τ* ≫ 1, generates a response which (see the derivation in Appendix G), up to corrections of the order of $1/\sqrt{K}\tau_{S}$, is given by Eq. (21) [dashed line in Fig. 6(b)]. Similarly to what happens in networks with instantaneous synapses and *a* ~ *K*^−*α*^, this finite response emerges because recurrent interactions set *μ* very close to threshold, at a distance $\theta -\mu \sim 1/\sqrt{K}$ that matches the size of the membrane potential fluctuations [Eq. (25), $\sigma \sqrt{\tau /\tau_{S}}\sim 1/\sqrt{K}$]. Hence, as the input to the network increases, recurrent interactions restore the drift-diffusion balance by adjusting the membrane potential mean *μ* close to threshold, so that fluctuations can sustain firing. Moreover, the single-neuron membrane potential correlation approaches *τ*_*S*_ and firing becomes bursty, with periods of regular spiking randomly interspersed in time.

**FIG. 7.**
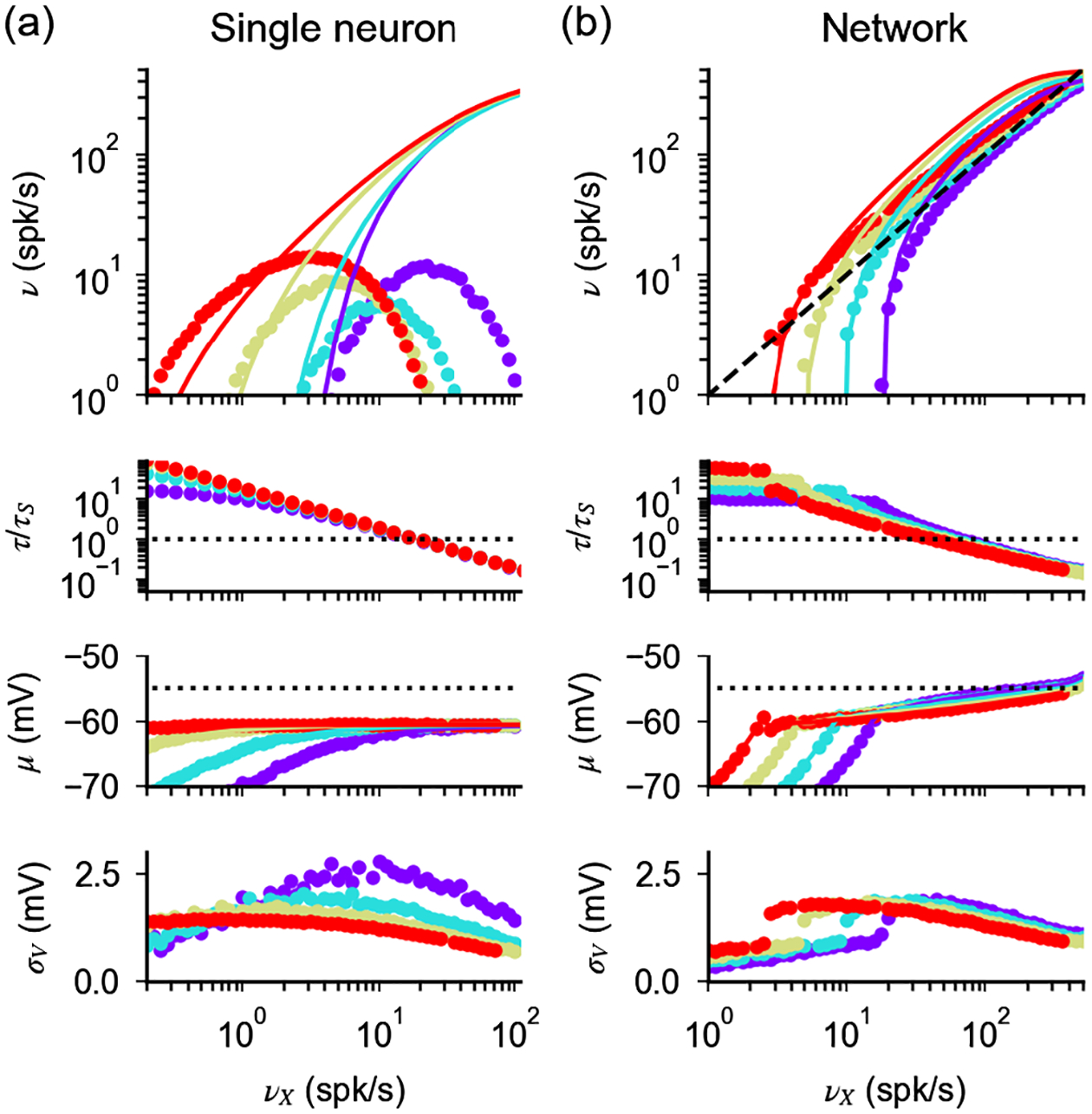
Single-neuron and network response with finite synaptic time constants, when both *a* and *τ*_*S*_ are rescaled with *K*. (a) Single-neuron response as a function of input rate *ν*_*X*_, computed numerically from Eqs. (1) and (23). Different colors correspond to different values of *K* (10^3^, purple; 10^4^, light blue; 10^5^, yellow; 10^6^, red) with *a* and *τ*_*S*_ scaled as in Eqs. (14) and (26); for *K* = 10^3^, *a* = 0.01 and *τ*_*S*_ = 1 ms (i.e., $\tau_{S}^{*}=10$ ms). The scaling relation described in the main text preserves the response properties observed in Fig. 6. (b) Analogous to (a) but in the network case; colors correspond to *K* = 500, 10^3^, 2 × 10^3^, and 4 × 10^3^. For *K* = 10^3^, *a* = 0.002 and *τ*_*S*_ = 1 ms.

We next discuss the effects of the values of *τ*_*S*_ and coupling strength on how the model response evolves with inputs; this discussion is relevant for both the single-neuron and the network model. In Appendix G, using existing analytical expansions [67,68,70,72] and numerical simulations, we show that neural responses obtained with finite *τ*_*S*_ are in good agreement with predictions obtained using a short synaptic time constant approximation for *τ*_*S*_/*τ* ≲ 0.1 and are captured by predictions obtained with a large synaptic time constant approximation for *τ*_*S*_/*τ* ≳ 1. The input value at which *τ*_*S*_/*τ* ~ 1, i.e., *ν*_*X*_ ~ 1/*aKτ*_S_, determines the input range over which the model expresses one of the two behaviors. Therefore, models with larger (smaller) *τ*_*S*_ or coupling strength have a smaller (larger) region of inputs in which their response is captured by results obtained with instantaneous synapses (Figs. 6 and 7). Importantly, when biologically relevant parameters are considered (e.g., Fig. 6), both the small and the large *τ*_*S*_/*τ* behaviors are expected to appear. In fact, biological synapses span a wide range of parameters, and most neuron types typically express both fast and slow synaptic receptors; in this condition, fast synapses (characterized by *τ*_*S*_ of a few milliseconds) are the ones that drive rapid membrane potential fluctuations and, hence, firing. Assuming *aK* ~ 10, we find that the transition from small to large *τ*/*τ*_*S*_ in the cortex is expected to appear for inputs *ν*_*X*_ ~ 1/*aKτ*_*S*_ ~ 10–100 spk/s, which is compatible with experimentally observed firing rates [23,46–54].

We next investigate if and under which conditions the input-dependent behavior described in this section is preserved in the strong-coupling limit. For large inputs, the membrane potential dynamics of Eq. (25) becomes independent of *a* for large *K*, and, hence, the model behavior is independent of the scaling relation used. For low inputs and finite coupling, the model behaves as in the case of instantaneous synapses, and, therefore, response properties can be preserved in the strong-coupling limit only if *a* ~ 1/ log(*K*). With this scaling, the value of *ν*_*X*_ separating the low and large input regimes decreases with coupling strength as log(*K*)/*Kτ*_*S*_. This is problematic because, as coupling increases, the model loses its low input behavior and converges to a pathological state in which, for all inputs, membrane potential fluctuations become small, the single-neuron response is suppressed, and, in the network case, the membrane potential is squeezed close to threshold. Thus, to preserve the input-dependent behavior in the strong-coupling limit, the synaptic time constant should decrease with coupling strength as

(26) $$\tau_{S}=\frac{\tau_{S}^{*}}{aK}\sim \frac{\text{log}(K)}{K},$$

where $\tau_{S}^{*}$ is a constant independent of *a* and *K*. In Fig. 7, we show that the scaling of Eq. (26) preserves the input-dependent response as coupling increases.

The activity-dependent drift-diffusion balance described here produces features that are not present in models with instantaneous synapses and that can be tested experimentally (see Table I for a summary). First, the increase of *μ* with inputs is absent in strongly coupled networks with instantaneous synapses and is consistent with the increased membrane potential observed in cortical circuits with the strength of sensory stimuli [23,49]. Second, with instantaneous synapses, the decay time constant of the autocorrelation of the membrane potential is of the order of *τ* and, hence, decreases, without bounds, as 1/*ν*_*X*_ with inputs. The finite synaptic time constant modifies the input dependence of the autocorrelation time constant—it decreases with *τ* for low inputs and becomes constant (of the order of *τ*_*S*_) for larger inputs. Third, with a finite synaptic time constant, firing becomes more bursty as input increases; this effect should be more prominent in networks with stronger coupling (e.g., prefrontal cortex). Fourth, synaptic dynamics makes the robustness of network response to connection heterogeneity input dependent: For small inputs, *τ*_*S*_/*τ* ≪ 1 and ${\text{CV}}_{K}^{*}\sim 1/\text{log}(K)$; for large inputs, *τ*_*S*_/*τ* ≫ 1 and ${\text{CV}}_{K}^{*}\sim 1/\sqrt{K}$ (derivation in Appendix G). Therefore, the model predicts that networks of neurons with heterogeneous connections and a log-normal distribution of rates for low inputs (e.g., Refs. [52–54]) should show an increasing number of silent and regular spiking cells as the input strength increases.

## IX. DISCUSSION

In this work, we analyzed networks of strongly coupled conductance-based neurons. The study of this regime is motivated by the experimental observation that in cortex *K* is large, with single neurons typically receiving inputs from thousands of presynaptic cells. We showed that the classical balanced state idea [5,6], which was developed in the context of current-based models and features synaptic strength of the order of $1/\sqrt{K}$ [7,8], results in current fluctuations of very small amplitude, which can generate firing in networks only if the mean membrane potential is extremely close to threshold. This is inconsistent with intracellular recordings in the cortex that show large membrane potential fluctuations (see, e.g., Refs. [21,46–51]). To overcome this problem, we introduced a new scaling relation which, in the case of instantaneous synaptic currents, maintains firing by preserving the balance of input drift and diffusion at the single-neuron level. Assuming this scaling, the network response automatically shows multiple features that are observed in the cortex *in vivo*: irregular firing, wide distribution of rates, membrane potential with non-negligible distance from threshold, and fluctuation size. When finite synaptic time constants are included in the model, we showed that these properties are preserved for low inputs but are gradually modified as inputs increase: The membrane mean approaches threshold, while its fluctuations decrease in size and develop non-negligible temporal correlations. These properties, which are summarized in Table I, provide a list of predictions that could be tested experimentally by analyzing the membrane potential dynamics as a function of the input strength in cortical neurons.

When synaptic time constants are negligible with respect to the membrane time constant, our theory shows properties that are analogous to those of the classical balanced state model: linear transfer function, CV of the order of one, and distribution of membrane potentials with finite width. However, these properties emerge from a different underlying dynamics than in the current-based model. In current-based models, the mean input current is at a distance of the order of one from threshold in units of input fluctuations. In conductance-based models, this distance increases with coupling strength, and firing is generated by large fluctuations at strong coupling. The different operating mechanism manifests itself in two ways: the strength of synapses needed to sustain firing and the robustness to connection heterogeneity, as we discuss in the next paragraphs.

The scaling relation determines how strong synapses should be to allow firing at a given firing rate, for a given value of *K*. In current-based neurons, irregular firing is produced as long as synaptic strengths are of the order of $1/\sqrt{K}$. In conductance-based neurons, stronger synapses are needed, with a scaling which approaches 1/ log (*K*) for large *K*. We showed that both scaling relations are in agreement with data obtained from culture preparations [63], which are limited to relatively small networks, and argued that differences might be important *in vivo*, where *K* should be larger.

In current-based models, the mean input current must be set at an appropriate level to produce irregular firing; this constraint is realized by recurrent dynamics in networks with random connectivity and strong enough inhibition [7–9]. However, in networks with structural heterogeneity, with connection heterogeneity larger than $1/\sqrt{K}$, the variability in mean input currents produces significant departures from the asynchronous irregular state, with large fractions of neurons that become silent or fire regularly [56]. This problem is relevant in cortical networks [56], where significant heterogeneity of in-degrees has been reported [50,57–62], and different mechanisms have been proposed to solve it [56]. Here, we showed that networks of conductance-based neurons also generate irregular activity without any need for fine-tuning and, furthermore, can support irregular activity with substantial structural heterogeneity, up to the order of 1/ log(*K*). Therefore, these networks are more robust to connection heterogeneity than the current-based model and do not need the introduction of additional mechanism to sustain the asynchronous irregular state.

When the synaptic time constant is much larger than the effective membrane time constant, we showed that, regardless of synaptic strength, the size of membrane potential fluctuations decreases and firing in the network is preserved by a reduction of the distance from threshold of the mean membrane potential. Moreover, the robustness to heterogeneity in connection fluctuations decreases substantially (the maximum supported heterogeneity becomes of the order of $1/\sqrt{K}$), and the membrane potential dynamics becomes correlated over a timescale fixed by the synaptic time constant. The network response at low rates is well approximated by that of networks with instantaneous synapses, and the regime of large synaptic time constant is reached gradually, as the input to the network increases (Fig. 6). This observation provides a list of predictions on how properties of cortical networks should evolve with input strength (summary in Table I.) that are testable experimentally. While some of these predictions require new experiments to be validated, we point out that one of them—that the equilibrium value of the membrane potential should increase with inputs—is consistent with the increased membrane potential observed in cortical circuits with the strength of sensory stimuli [23,49].

In conductance-based models, we showed that response properties observed at finite coupling survive in the strong-coupling *K* → ∞ limit only if unitary conductances obey a specific scaling law [Eq. (14)], and synaptic time constants also obey a scaling law [Eq. (26)]. While there is evidence in cortical cultures that average synaptic strengths do decay with increasing connectivity [63], no such evidence exists to our knowledge to support decreasing synaptic time constants with increasing connectivity. However, it is well known that synaptic decay time constants depend on subunit composition of the receptors (see, e.g., Ref. [73] for GABA receptors, Ref. [74] for NMDA receptors, and Ref. [75] for AMPA receptors), and subunit composition can depend on synaptic activity (e.g., Ref. [76]). It is thus tempting to speculate that both scaling laws could be implemented in neurobiological circuits. If such plasticity exists, our theory predicts that it should produce smaller synaptic time constants in networks with larger *K*.

In our analytical calculations, we have neglected correlations between neurons and assumed the network operates in the asynchronous regime. This assumption is consistent with observations that correlations between cells in cortex *in vivo* can in some cases be small, i.e., of the order of 0.01 [77,78]. It is also consistent with the results of our numerical simulations, which show good agreement with the calculations in networks with connection probabilities of 0.1, on the same order of magnitude as observed connection probabilities in cortex. However, correlations between neurons can vary significantly between cortical state, layer, and firing rate, with many studies finding average correlation coefficients of the order of 0.1 or more (e.g., Ref. [79]). Intriguingly, weak but nonzero correlations between inputs, on the order of 0.1, have been argued to be necessary to quantitatively capture the amplitude of membrane potential fluctuations observed in the cat cortex [21]. Understanding how correlations affect the results obtained in our work is an important problem which should be addressed in the future.

Experimental evidence suggests that the response to multiple inputs in cortex is nonlinear (for an overview, see Ref. [80]). Such nonlinearities, which are thought to be fundamental to perform complex computations, cannot be captured by the classical balanced state model, as it features a linear transfer function [7,8]. Several studies have shown how relaxing assumptions underlying the classical balanced state model can lead to nonlinear responses. In particular, moderate coupling and power-law single-neuron input-output function [80–82], short-term plasticity [83], and differential inputs to subsets of excitatory neurons [84] can lead to nonlinearities. We have recently shown [85] that nonlinear responses appear in networks of current-based spiking neurons when coupling is moderate and only at response onset or close to single-neuron saturation. Here, we have shown that response onset and saturation nonlinearities appear also in networks of conductance-based neurons when coupling is moderate. In addition, we have found that synaptic time constants provide an additional source of nonlinearity, with nonlinear responses emerging as the network transitions between the response onset and saturation. A full classification of the nonlinearities generated in these networks is outside the scope of this work but could be performed by generalizing the approach developed in Ref. [85].

The strength of coupling in a network, both in the current-based model [81,85] and in the conductance-based model (e.g., Fig. 3), determines the structure of its response and, hence, the computations it can implement. Recent theoretical work, analyzing experimental data in the framework of current-based models, has suggested that the cortex operates in a regime of moderate coupling [82,86], where response nonlinearities are prominent. In conductance-based models, the effective membrane time constant can be informative on the strength of coupling in a network, as it decreases with coupling strength. Results from *in vivo* recordings in the cat parietal cortex [21] showed evidence that single-neuron response is sped up by network interactions. In particular, measurements are compatible with inhibitory conductance approximately 3 times larger than leak conductance and support the idea that the cortex operates in a “high-conductance state” [22]. This limited increase in conductance supports the idea of moderate coupling in cortical networks, in agreement with what was found in previous work [82,86]. More recent studies have, however, obtained results that seem at odds with the high-conductance state idea. Recent whole cell recordings have reported that an intrinsic voltage-gated conductance, whose strength decreases with membrane potential, contributes to the modulation of neuronal conductance of cells in the primary visual cortex of awake macaques and anesthetized mice [23]. For spontaneous activity, this intrinsic conductance is the dominant contribution to the cell conductance and drives its (unexpected) decrease with increased depolarization. For activity driven by sensory stimuli, on the other hand, modulations coming from synaptic interactions overcome the effect of the intrinsic conductance, and neuronal conductance increases with increased depolarization. The decrease in conductance observed during spontaneous activity in Ref. [23] seems incompatible with previous experimental results [22], and it is still unclear which differences between experimental preparations underlie these differences. While a resolution of this discrepancy will require additional experimental work, we point out that our work is relevant for both scenarios. In fact, our analysis shows that voltage-dependent currents, such as that produced by the voltage-gated channels [23] or during spike generation [44], affect quantitatively, but not qualitatively, the single-neuron response. Moreover, our theory explains the mechanisms shaping response properties at finite coupling and identifies a scaling relation that preserves these properties in the strong-coupling limit. Therefore, results described in this contribution seem to be a general property of networks of spiking neurons with conductance-based synapses, and they should be relevant for a wide range of single-neuron models and coupling strengths.

Understanding the dynamical regime of operation of the cortex is an important open question in neuroscience, as it constrains which computations can be performed by a network [81]. Most of the theories of neural networks have been derived using rate models or current-based spiking neurons. Our work provides theoretical tools to investigate the dynamics of strongly coupled conductance-based neurons, and it suggests predictions that could be tested experimentally.

## Figures and Tables

**TABLE I.** Overview of networks of current-based and conductance-based neurons. The synaptic time constant strongly affects response properties in networks of conductance-based neurons. Properties similar to what is observed in the cortex emerge in these networks if *a* ~ 1/ log *K* and input rates are lower than or comparable to $1/\tau_{S}^{*}$ [defined in Eq. (26)]. The model predicts that response properties should gradually mutate as the input to the network increases and, for large inputs, should coincide with those indicated in the last line of the table. In the table, the different quantities related to the membrane potential represent the mean distance from threshold (*θ* − *μ*), the size of temporal fluctuations (*σ*_*V*_), and the membrane potential correlation time constant (*τ*_*V*_).

Synaptic model	Ratio of synaptic and membrane time constant (*τ*_*S*_/*τ*)	Synaptic strength	Membrane potential statistics	Activity structure	Heterogeneity of in-degree supported (${\text{CV}}_{K}^{*}$)
Current-based (balanced state model)	Constant, independent of *v*_*X*_, *a*, and *K*	$J\sim (1/\sqrt{K})$	*θ* – *μ ~ σ*_*V*_ ~ 1; *τ*_*V*_ *~ τ*_*L*_	Irregular firing, CV of ISI ~ 1	$\sim (1/\sqrt{K})$
Conductance-based	≪ 1 for *v*_*X*_ ≪ (1/*τ*_S_*); always satisfied for instantaneous synapses ($\tau_{S}^{*}=0$)	*a* ~ (1/ log *K*)	*θ* – *μ ~* 1; $\sigma_{V}\sim (1/\sqrt{\text{log }K})$; *τ*_*V*_ ~ log(*K*)/*K*	Irregular firing, CV of ISI ~ 1	~ (1/ log *K*)
		*a* ~ *K*^−*α*^, *α* > 0	*θ* – *μ ~ σ*_*V*_ ~ *K*^(−*α*/2)^; *τ*_*V*_ ~*K*^*α*−1^	Irregular firing, CV of ISI ~ 1	~*K*^−*α*^
	≫ 1 for *v*_*X*_ ≫ (1/*τ*_S_*)	Any scaling	$\theta -\mu \sim \sigma_{V}\sim (1/\sqrt{K})$; *τ*_*V*_ *~ τ*_*L*_	Irregular bursting	$\sim (1/\sqrt{K})$

**FIG. 8.**
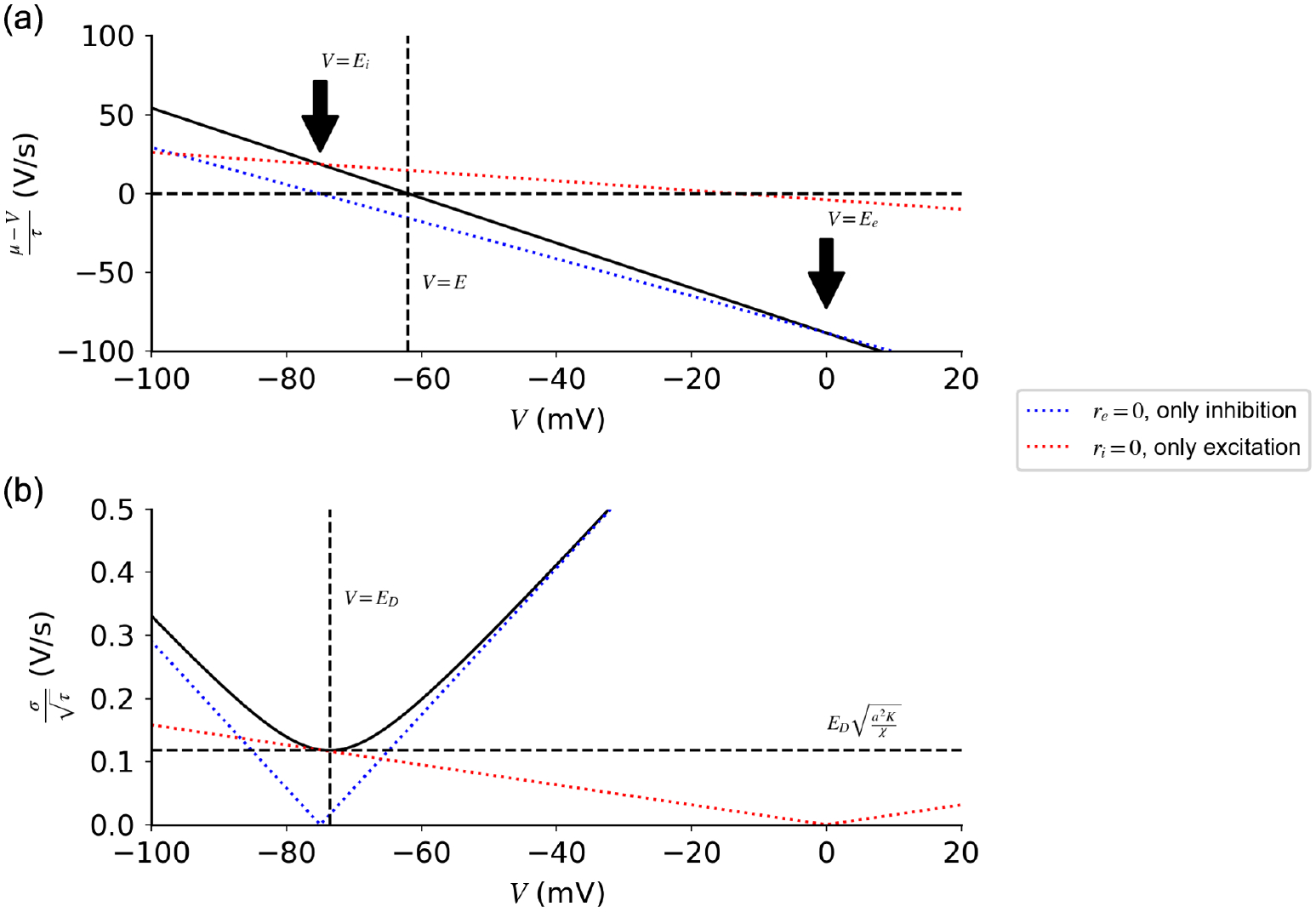
Drift and diffusion terms of Eq. (4) as a function of the voltage. (a) Input drift as a function of membrane potential *V* produced with both inhibitory and excitatory inputs (black line), excitatory inputs only (red dotted line), or inhibitory inputs only (blue dotted line). The drift term decreases monotonically with *V*, and it is zero at *V* = *μ*, which is a stable fixed point of the deterministic dynamics. (b) The noise variance is quadratic in *V*. Its minimum at $V=E_{S}$ is equal to $E_{D}\sqrt{a^{2}K/\chi }$. Note that the minimum amplitudes of drift and variance are obtained at different values of *V*.

**FIG. 9.**
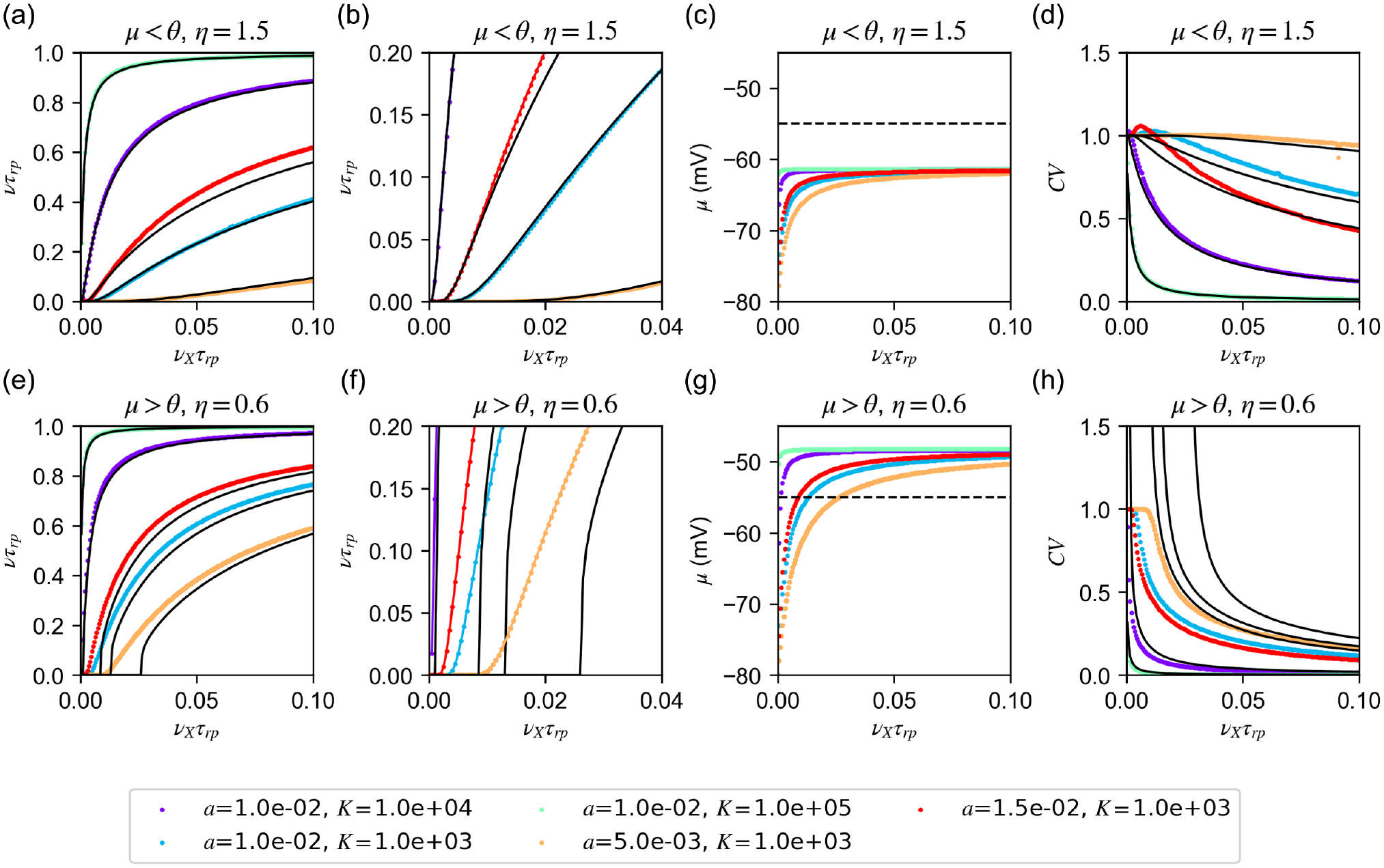
Response of a single conductance-based neuron to noisy inputs. Estimates of firing rate [(a),(b),(e),(f)], *μ* [(c),(g)], and CV [(d),(h)] obtained with numerical integration of Eqs. (A13), (13), and (A14) for different values of *a* and *K* (colored dots). For the two regimes *μ* < *θ* (first row) and *μ* > *θ* (second row), the transfer function saturates as *K* increases. Note that the same change in *a* has a more drastic effect if *μ* < *θ*; this is due to the exponential dependence that appears in Eq. (B6). The approximated expressions (continuous lines) capture the properties of the transfer function [(a) Eq. (B6) and (e) Eq. (B4)] and CV [(c) Eq. (B17) and (g) Eq. (B9)]. For small inputs (f), Eq. (B4) fails to describe the transfer function for some values of *K*, because the corresponding *μ* is below threshold. Simulations parameter are *g* = 12, *γ* = 1/4, and *η* = 1.5 (top) or 0.6 (bottom).

**FIG. 10.**
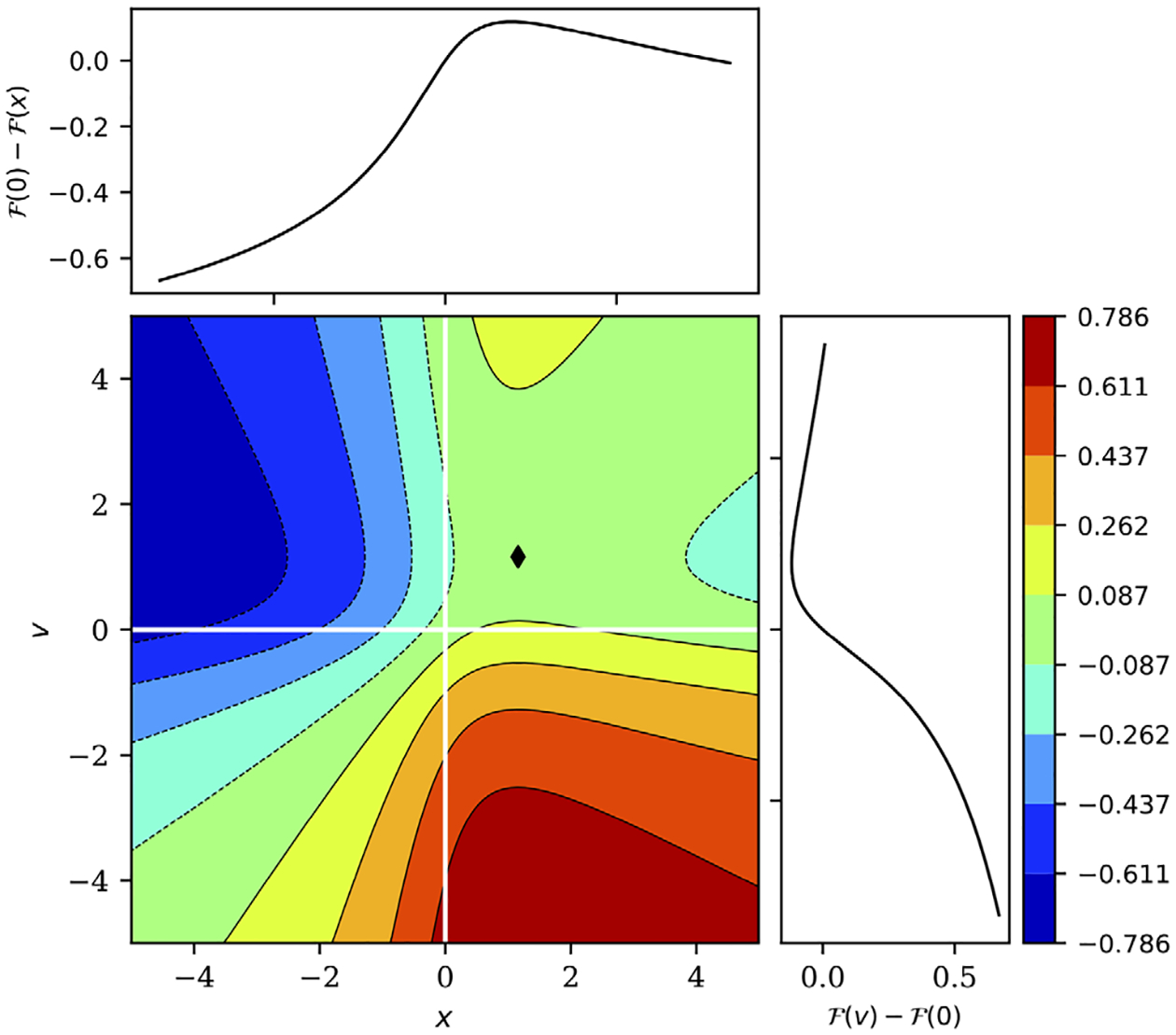
Graphical representation of the exponent in Eq. (A13). The function F(v)−F(x) is stationary at *x* = *v* = *α*; this point is a maximum for *x* and a minimum for *v*. Parameters are as in Fig. 8. In this figure, *α* = 1.2 (black diamond).

**FIG. 11.**
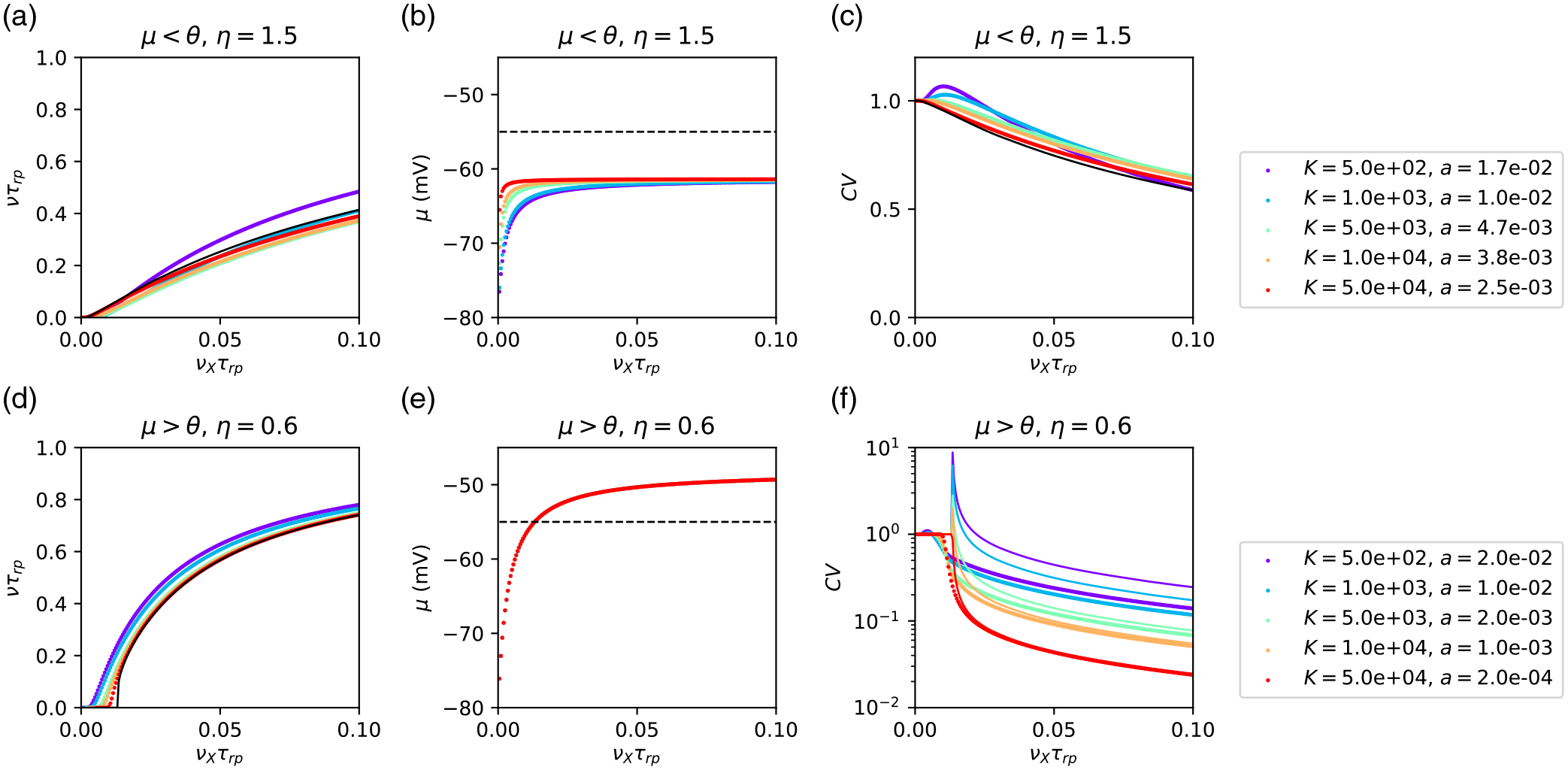
Scaling relationships preserving firing in the large *K* limit. Colored dots represent mean field transfer function [(a),(b)], CV [(c),(d)], and membrane potential [(e),(f)] obtained from Eqs. (A13), (A14), and (A8), respectively. Different colors correspond to different values of *a* and *K* which are scaled according to Eqs. (B22) (first row) and (B21) (second row). Mean field predictions are well described by the relevant approximated expressions (continuous lines). For *μ* < *θ*, the transfer function and CV are described by Eqs. (B22) (a) and (B17) (c); both quantities are invariant as *K* increases. For *μ* > *θ*, the transfer function and CV are described by Eqs. (B21) (a) and (B9) (c); note that, as explained in the text, the firing is preserved while the CV becomes smaller as *K* increases (different line colors correspond to different values of *K*). Parameters: *g* = 12 and *γ* = 1/4.

**FIG. 12.**
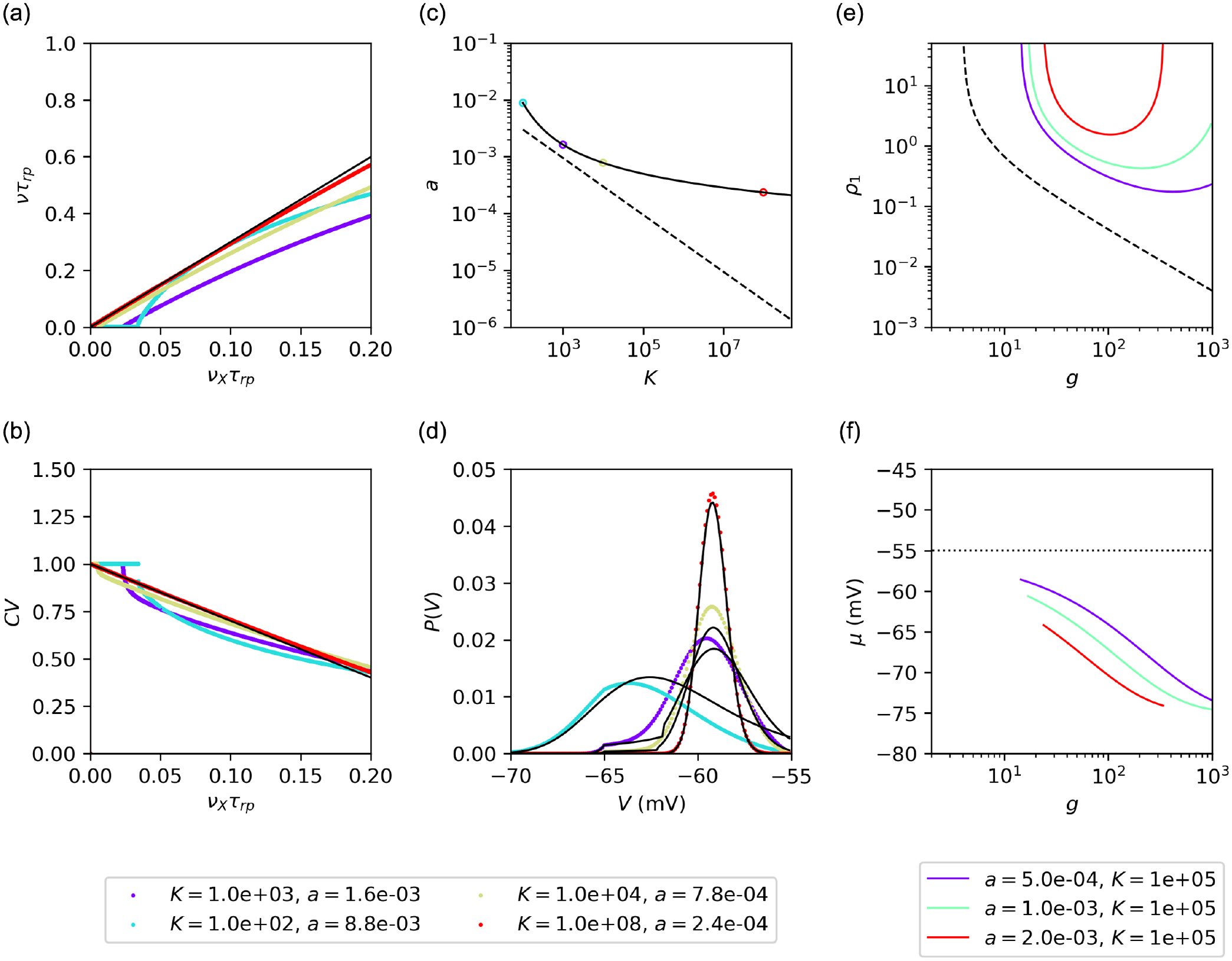
Strong-coupling limit of networks of conductance-based neurons in model *A*. Numerically computed network transfer function (a), CV (b), and probability distribution of the membrane potential (d) obtained from Eqs. (D4), (A12), and (B17). Different colors correspond to different values of *a* and *K* which are changed according to the scaling relation (D10) (c). As *K* increases the network transfer function and CV converges to the expression derived in the main text (black lines). Note that, unlike the case of a single neuron, the network transfer functions become linear. The probability distribution of the membrane potential becomes Gaussian and slowly converges to a delta function. (e) and (f) show the network gain and membrane potential, respectively, for different values of *a* at fixed *K*. Note that, unlike what happens in current-based networks (black dashed lines), the gain is not monotonic with *g*. Simulation parameters are as in Fig. 9; in (a)–(d), *g* = 20.

**FIG. 13.**
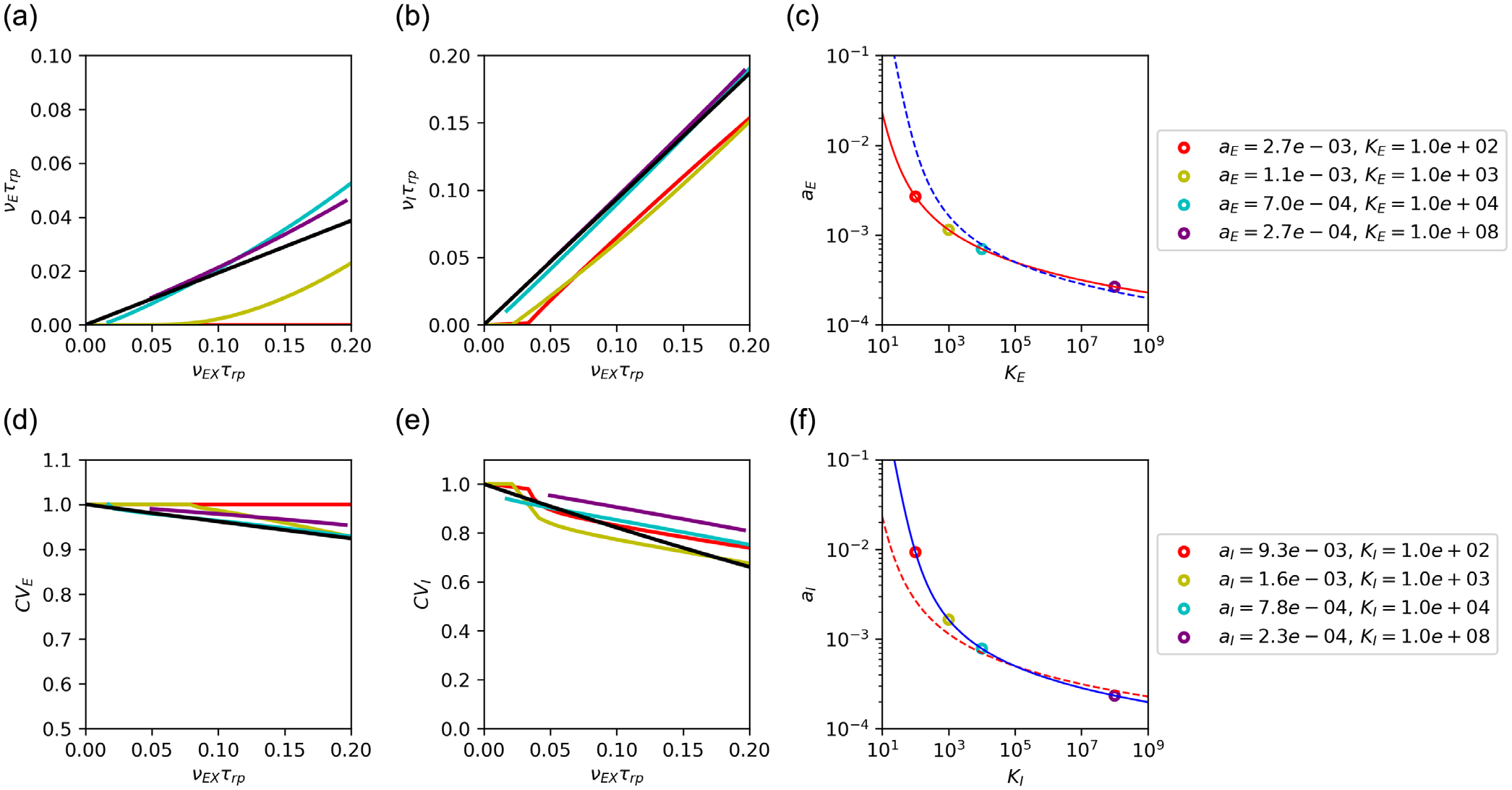
Limit of large *K* for networks, model *B*. The firing rate and CV of excitatory and inhibitory neurons in a network predicted by the mean field model for different values of inputs and *K*; the expected asymptotic behavior is shown in black. On the left [(c),(f)], we show the corresponding scaling relations with dots associated to the connectivity parameters. Simulations parameter: The two populations have *g*_*e*_ = 20.0 and *g*_*i*_ = 19.0; for both populations, *a* = 0.0005 for *K* = 10^5^; other parameters are as in Fig. 9.

**FIG. 14.**
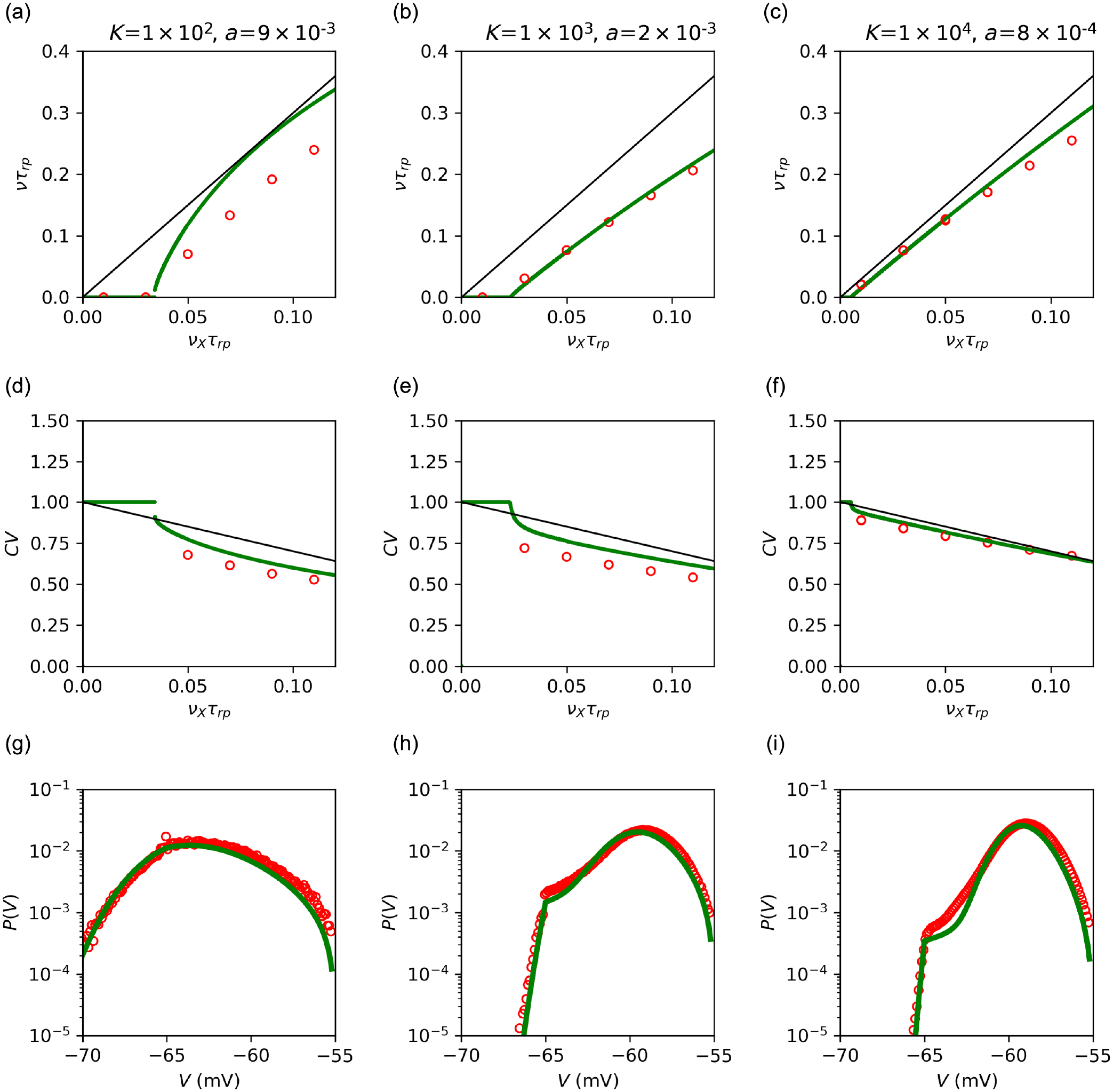
Comparison of mean field theory and numerical simulations. Network transfer function (first row), CV of ISI distribution (second row), and probability distribution of the membrane potential at *ν*_*E*_ = 0.05*τ*_*rp*_ (third row). In each, we show the mean field prediction (green), results from numerical simulations (red), and the value expected in the strong-coupling limit (black). Different columns correspond to different values of *K* and *a* which are scaled according to Eq. (D10). The agreement between network simulations (red) and mean field predictions (green) improves as *a* decreases, as expected, since we use the diffusion approximation to derive the results. Simulation parameters are *g* = 20 and *N*_*E*_ = *N*_*I*_ = *N*_*EX*_ = *N*_*IX*_ = 100*K*.

**FIG. 15.**
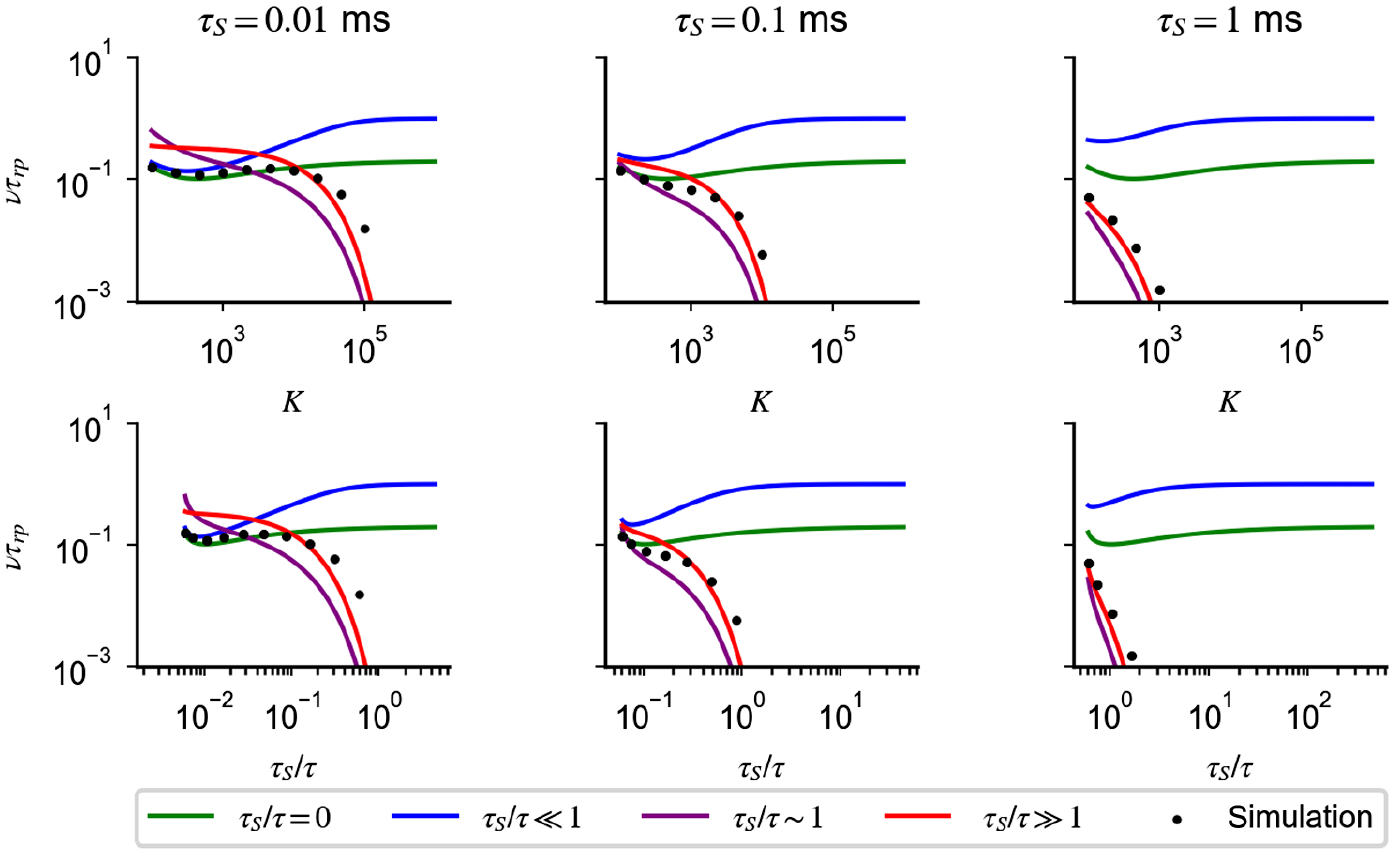
Synaptic time constant suppresses single-neuron response in the strong-coupling limit. Single-neuron response for different values of *K*, with *a* rescaled according to Eq. (14). Rates are plotted as a function of *K* (first row) and *τ*_*S*_/*τ* (second row); different columns correspond to different synaptic time constant *τ*_*S*_ (title). As *K* increases, because of the synaptic time constant *τ*_*S*_ non-negligible compared to the membrane time constant *τ*, rates computed numerically from Eq. (G1) (black dots) depart from the prediction of Eq. (10) (green). The dependency of the rate on *K* is captured by Eq. (G9) (blue) for small values of *τ*_*S*_/*τ* and by Eq. (G11) (red) for large values of *τ*_*S*_/*τ*. This decay cannot be prevented by a new scaling relation of *a* with *K* and provides an upper bound to how much coupling can be increased while preserving response. Simulations parameter: *a* = 0.006 for *K* = 10^3^, *g* = 12, and *η* = 1.46.

**FIG. 16.**
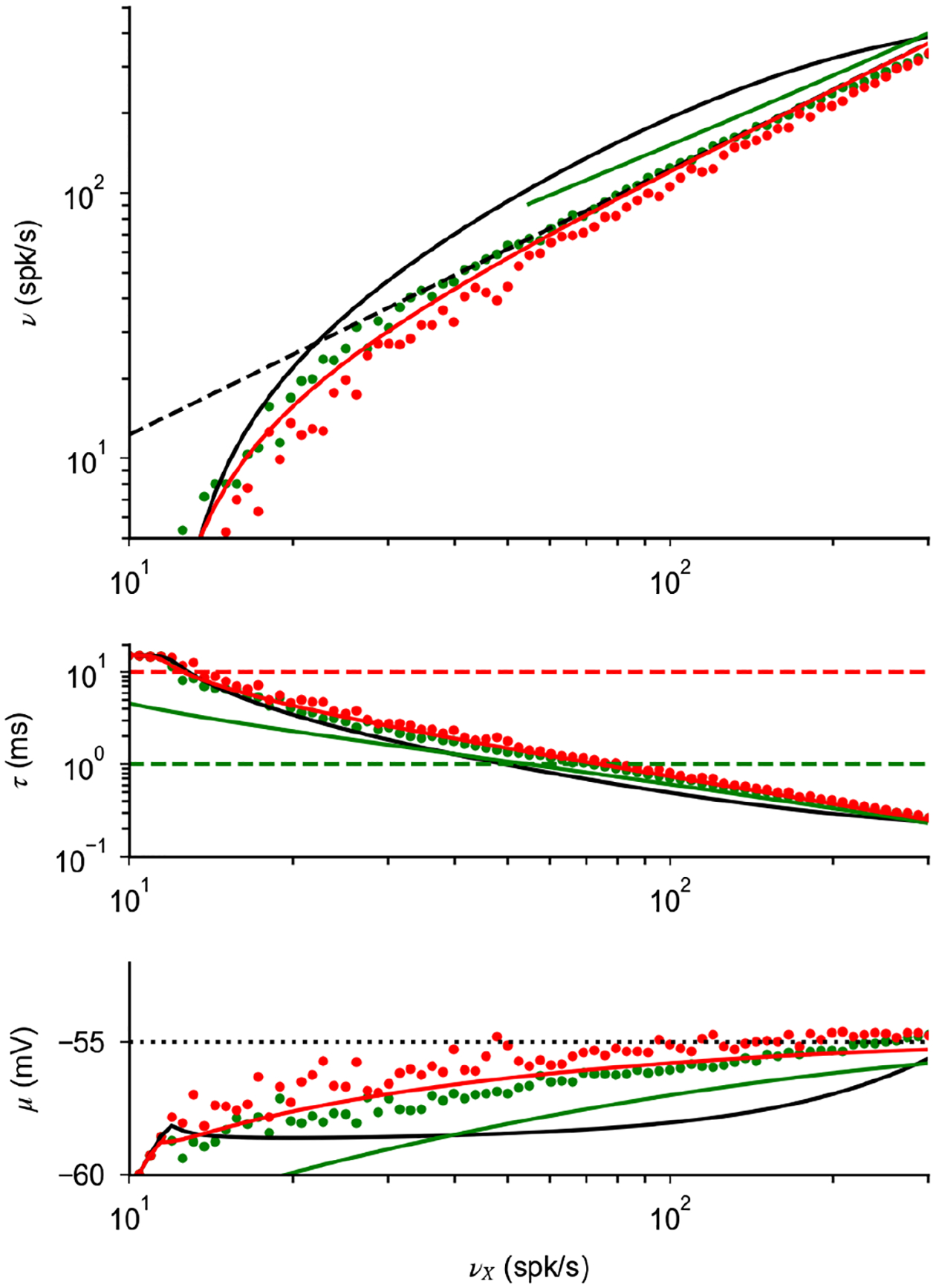
Approximation of network response for large *τ*_*S*_/*τ*. Plots analogous to Figs. 6(b) and 6(c) of the main text. Dots represent network response as a function of input rate *ν*_*X*_, computed numerically from Eqs. (1) and (23) for *τ*_*S*_ = 1 ms (green) and *τ*_*S*_ = 10 ms (red). Continuous lines correspond to the prediction obtained with instantaneous synapses (black) and for large synaptic time constant [Eqs. (G12), (5), and (G13), colored lines]. As explained in the text, the latter predictions are valid only for large *τ*_*S*_/*τ*; because of this, we plot only values obtained for *τ*_*S*_/*τ* > 1. For *τ*_*S*_/*τ* ≫ 1, the network response is well described by Eq. (21) of the main text.

**FIG. 17.**
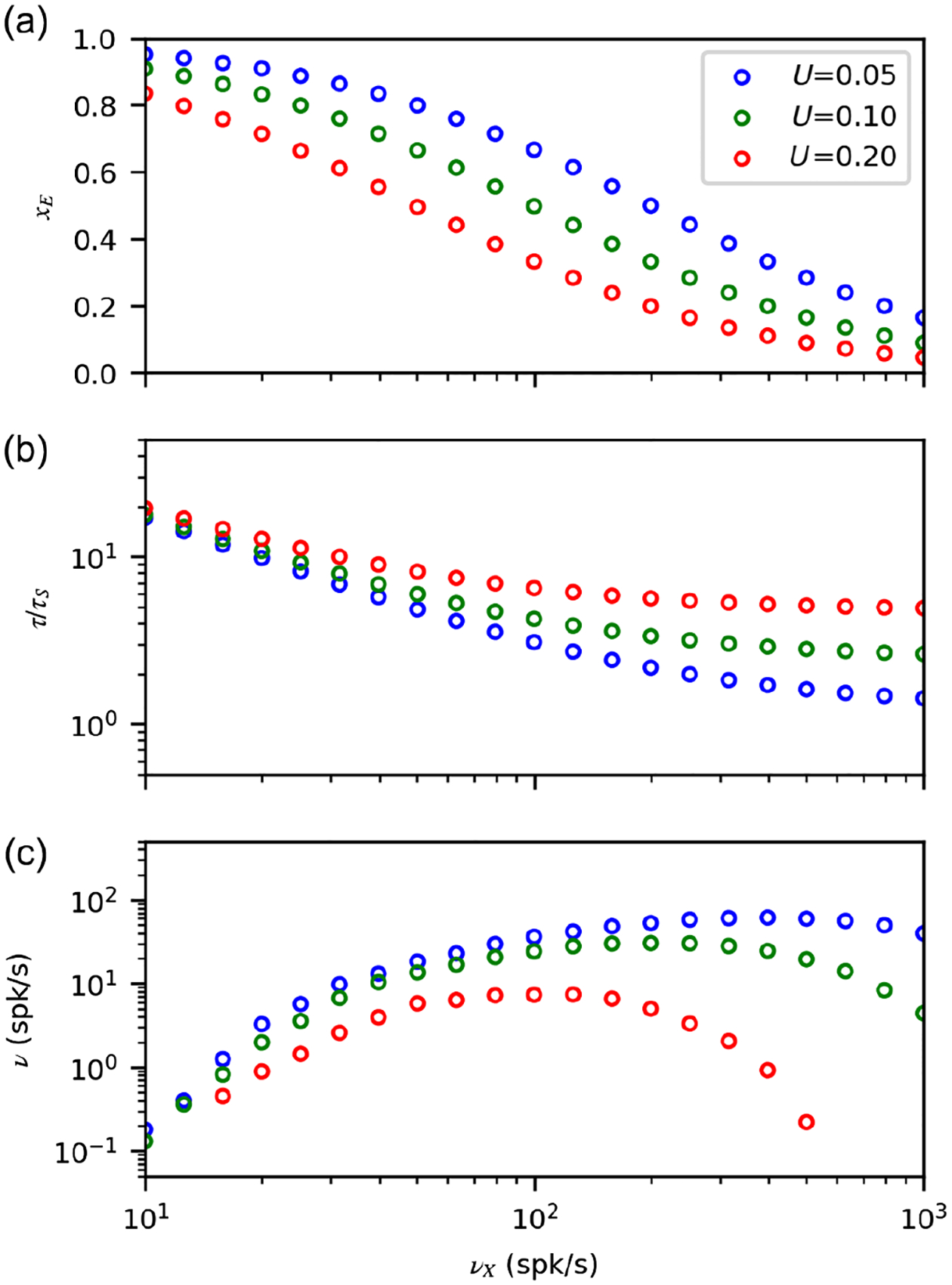
Effect of short-term plasticity on single-neuron response. (a) Dependence of depression variable *x*_*E*_ on the presynaptic rate given by Eq. (H4), for different values of *U*. (b) Effective membrane time constant *τ*_*X*_ as a function of *ν*_*X*_, with depression variable as in (a). Short-term plasticity limits the decrease of *τ* with inputs. (c) Firing rate of conductance-based neurons computed numerically as in Fig. 6(a), but with depression variable as in (a). Even with short-term plasticity, single-neuron response is suppressed for large *ν*_*X*_. Parameters are *τ*_*D*_ = 100 ms, *K* = 10^3^, *a* = 0.01, *τ*_*S*_ = 0.1 ms, *g* = 12, *η* = 1.4, and *γ* = 1/4.

## APPENDIX A: CALCULATIONS IN THE MULTIPLICATIVE NOISE CASE

In the main text, we analyze the distribution of membrane potential, firing rate, and CV using the effective time constant approximation, which neglects the dependence of the noise amplitude on the membrane potential. This approximation is motivated by the fact that corrections to this approximation are of the same order of shot noise corrections to the diffusion approximation used to describe synaptic inputs [87]. In this section, we derive results without resorting to the effective time constant approximation (i.e., keeping the voltage dependence of the noise term) and show that the results derived in the main text remain valid, even though it complicates the calculations. The inclusion of shot noise corrections is outside the scope of this contribution.

### 1. Equations for arbitrary drift and diffusion terms

In this section, we compute the probability distribution of the membrane potential, the firing rate, and the CV of ISI of a neuron whose membrane potential follows the equation

(A1)
$$\frac{dV}{dt}=A(V)+B(V)\zeta .$$

Equation (4) of the main text is a special form of Eq. (A1) with

(A2)
$$A(V)=\frac{\mu -V}{\tau },\;B(V)=\frac{\sigma (V)}{\sqrt{\tau }}.$$

The Fokker-Plank equation associated to Eq. (A1), in the Stratonovich regularization scheme, is given by

$$\frac{dP}{dt}=-\frac{\partial J}{\partial V},$$

where *P* is the probability of finding a neuron with membrane potential *V* and *J* is the corresponding probability current given by

(A3)
$$J=\left(A+\frac{1}{2}B\frac{\partial B}{\partial V}\right)P-\frac{1}{2}\frac{\partial B^{2}P}{\partial V}.$$

We are interested in the stationary behavior of the system in which *P* does not depend on time and the current *J* is piecewise constant. In particular, for *V* between the activation threshold *θ* and the resting potential *V*_*r*_, *J* is equal to the neuron firing rate *ν*, and the normalization condition implies

$$\int_{V_{r}}^{\theta }P(V)dV+\nu \tau_{rp}=1,$$

where *τ*_*rp*_ is the refractory period.

To derive the probability distribution of the neuron potential, we introduce in Eq. (A3) the integrating factor

$$W(V)=\text{exp}\left[-2\int^{V}du\frac{A(u)+\frac{1}{2}B(u)\frac{\partial B(u)}{\partial u}}{B{\left(u\right)}^{2}}\right]$$

and obtain

$$-2\nu W(V)\theta \left(V-V_{r}\right)=\frac{\partial }{\partial V}\left[W(V)B{\left(V\right)}^{2}P(V)\right].$$

Using the boundary condition *P*(*θ*) = 0, we find

(A4)
$$P(V)=\frac{2\nu }{W(V)B{\left(V\right)}^{2}}\int_{V}^{\theta }duW(u)\theta \left(u-V_{r}\right)$$

and

$$\frac{1}{\nu }=\tau_{rp}+2\int_{-\infty }^{\theta }dx\frac{1}{W(x)B{\left(x\right)}^{2}}\int_{x}^{\theta }duW(u)\theta \left(u-V_{r}\right).$$

Integrating by parts, we obtain

(A5)
$$\frac{1}{\nu }=\tau_{rp}+2\int_{V_{r}}^{\theta }dvW(v)\int_{-\infty }^{v}dx\frac{1}{W(x)B{\left(x\right)}^{2}}.$$

This solution is obtained in general form in Ref. [41] and for the specific form of Eq. (A2) in Ref. [11].

We now compute the coefficient of variation of the interspike interval. The moments *T*_*k*_ of the interspike intervals of the stochastic process defined by Eq. (A1) are given by (see Ref. [43])

$$\frac{B{\left(x\right)}^{2}}{2}\frac{d^{2}T_{k}(x)}{dx^{2}}+\left(A(x)+\frac{1}{2}B(x)\frac{\partial B(x)}{\partial x}\right)\frac{dT_{k}(x)}{dx}=-kT_{k-1}(x)$$

with boundary conditions

$$T_{k}(\theta )=0,\;\frac{dT_{k}(b)}{dx}=0;$$

i.e., *θ* is an absorbing boundary and *b* is a reflective boundary (we then consider the limit *b* → −∞). The general solution of an equation of the form

(A6)
$$\frac{d^{2}f(x)}{dx^{2}}+P(x)\frac{df(x)}{dx}=Q(x)$$

is

$$f(x)=\int_{\theta }^{x}dt\int_{-\infty }^{t}dzQ(z)\text{ exp }\left(\int_{t}^{z}dwP(w)\right).$$

For *T*_1_(*x*), we have

$$P(x)=\frac{2A(x)+B(x)\frac{\partial B(x)}{\partial x}}{B{\left(x\right)}^{2}},\;Q(x)=-\frac{2}{B{\left(x\right)}^{2}}.$$

For *T*_2_(*x*), we look for a solution of the form

$$T_{2}(x)=T_{1}{\left(x\right)}^{2}+R(x)$$

and find that *R* obeys an equation of the form of Eq. (A6) with

$$P(x)=\frac{2A(x)+B(x)\frac{\partial B(x)}{\partial x}}{B{\left(x\right)}^{2}},\;Q(x)=-2{\left(\frac{dT_{1}(x)}{dx}\right)}^{2}.$$

Combining the previous results, the CV of ISI is obtained as

(A7)
$${\text{CV}}^{2}=\frac{R(x)}{T_{1}{\left(x\right)}^{2}};$$

the explicit expression of the CV is given in the following section.

Equations (17), (10), and (15) of the main text are obtained from Eqs. (A4), (A5), and (A7), respectively, using Eq. (A2).

### 2. Equations for conductance-based LIF neurons

Starting from Eqs. (4) and (5) of the main text, we write the different terms as

(A8)
$$\tau^{-1}=\tau_{L}^{-1}+aK\omega^{-1},\;\mu =\tau \left\{E_{L}/\tau_{L}+aK\left[r_{E}E_{E}+r_{I}g\gamma E_{I}\right]\right\},\;\sigma^{2}=a^{2}K\frac{\tau }{\chi }\left[{\left(V-E_{S}\right)}^{2}+E_{D}^{2}\right],$$

where, to shorten the expressions, we introduce two auxiliary variables with time dimension:

(A9)
$$\omega^{-1}=r_{E}+r_{I}g\gamma ,\;\chi^{-1}=r_{E}+r_{I}g^{2}\gamma ,$$

as well as two variables with voltage dimensions:

(A10)
$$E_{S}=\chi \left(r_{E}E_{E}+r_{I}g^{2}\gamma E_{I}\right),\;E_{D}=\chi \sqrt{r_{E}r_{I}g^{2}\gamma }\left(E_{E}-E_{I}\right).$$

The terms −(*V* – *μ*)/*τ* and $\sigma (V)\zeta /\sqrt{\tau }$ in Eq. (4) represent the input drift and noise to the membrane dynamics, respectively. The voltage dependence of these terms is sketched in Fig. 8.

In the large *K* limit, the different terms in Eq. (4) scale as

(A11)
$$\tau \sim \frac{\omega }{aK},\;\mu \sim \omega \left(r_{E}E_{E}+r_{I}g\gamma E_{I}\right),\;\sigma \sqrt{\tau }\sim \frac{\omega }{\sqrt{\chi K}}\sqrt{{\left(V-E_{S}\right)}^{2}+E_{D}^{2}};$$

while the values of *ω*, *μ*, $E_{S}$, and $E_{S}$ are independent of *K*. It follows that the noise term $\sigma \sqrt{\tau }$ and the time constant *τ* in Eq. (4) become small in the strong-coupling limit. This result is analogous to what we obtain in the main text with the effective time constant approximation, since this approximation does not change how these terms scale with *a* and *K*.

We now insert the drift and diffusion terms of the conductance-based LIF neuron in Eqs. (A4), (A5), and (A7) and obtain

(A12)
$$P(V)=\frac{2\nu \chi E_{D}e^{-F(V)/a}}{a^{2}K\left[{\left(V-E_{S}\right)}^{2}+E_{D}^{2}\right]}\int_{u(V)}^{v_{\text{max}}}dx\theta \left[x-u\left(V_{r}\right)\right]e^{F(x)/a},$$

(A13)
$$\frac{1}{\nu }=\tau_{rp}+\frac{2\chi }{a^{2}K}\int_{v_{\text{min}}}^{v_{\text{max}}}dv\int_{-\infty }^{v}dx\frac{1}{x^{2}+1}\text{exp}\left[\frac{F(v)-F(x)}{a}\right],$$

and

(A14)
$${\text{CV}}^{2}=\frac{8\chi^{2}\nu^{2}}{a^{4}K^{2}}\int_{v_{\text{min}}}^{v_{\text{max}}}dv\int_{-\infty }^{v}dz\text{exp}\left[\frac{F(v)-F(z)}{a}\right]\times {\left\{\int_{-\infty }^{z}dw\frac{1}{w^{2}+1}\text{exp}\left[\frac{F(z)-F(w)}{a}\right]\right\}}^{2},$$

where

(A15)
$$F(x)=\frac{2\chi }{aK\tau }\left[\frac{1}{2}\left(1-\frac{a^{2}K\tau }{2\chi }\right)\text{ log}\left(x^{2}+1\right)-\alpha \text{arctan}(x)\right],\;u(V)=\frac{V-E_{S}}{E_{D}},\;v_{\text{min}}=u\left(V_{r}\right),\;v_{\text{max}}=u(\theta ),\;\alpha =u(\mu ).$$

Equations (A12) and (A13) are analogous to those derived in Ref. [11]. To simplify the following analysis, we neglect the contribution of the term *a*^2^*Kτ*/2*χ*, which derives from the regularization scheme. This assumption is justified by the fact that, for large *K*, *τ* ~ 1/*aK* and the factor *a*^2^*Kτ*/2*χ* is of the order of *a* ≪ 1.

## APPENDIX B: CALCULATIONS IN THE STRONG-COUPLING REGIME—SINGLE NEURONS

In the main text, we derive a simplified expression for the single-neuron response neglecting the dependency of noise on membrane potential. In this section, we generalize this result to the case in which the full noise expression is considered. We compute simplified expressions of the single-neuron transfer function and CV, in both the subthreshold regime *μ* < *θ* and the suprathreshold regime *μ* > *θ*. These expressions are validated numerically in Fig. 9 and used in the last part of this section to define a scaling relation between *a* and *K* which preserves single-neuron firing in the strong-coupling limit.

### 1. Single-neuron transfer function at strong coupling

The starting point of our analysis is the observation that the integrand in Eq. (A13) depends exponentially on 1/*a* ≫ 1. This suggests to perform the integration with a perturbative expansion of the exponent. We show below that, since the exponent has a stationary point at *x* = *v* = *α* (see Fig. 10), the integration gives two qualitatively different results if *α* is larger or smaller than the upper bound of the integral *v*_max_. Moreover, since the condition *α* ≶ *v*_max_ corresponds to *θ* ≶ *μ*, the two behaviors correspond to supra- and subthreshold regimes, respectively.

#### a. Suprathreshold regime *v*_max_ *< α* (*θ* < *μ*)

The exponent in Eq. (A13) is negative for every value of *x*, except for *x* = *v*, in which it is zero. The integral in *x* can be written as

(B1)
$$I=\int_{-\infty }^{v}dxg(x)e^{f_{v}(x)/a}\;=\int_{-\infty }^{v}dxg(x)e^{(1/a)\left[f_{v}^{\prime }(v)(x-v)+f_{v}^{\prime \prime }(v)/2{\left(x-v\right)}^{2}+\cdots \right]}.$$

With a change of variable *z* = (*x* − *v*)/*a*, we obtain

(B2)
$$I=a\int_{-\infty }^{0}dzg(v+az)e^{f_{v}^{\prime }(v)z+af_{v}^{\prime \prime }(v)\left(z^{2}/2\right)+\cdots }.$$

Neglecting all the terms of the order of *a*, we get

(B3)
$$I=a\frac{g(v)}{f_{v}^{\prime }(v)}.$$

Performing the integration in *v*, we obtain

(B4)
$$\frac{1}{\nu }=\tau_{rp}+\tau \text{ log}\left(\frac{\mu -V_{r}}{\mu -\theta }\right).$$

Equation (B4) is the transfer function of a deterministic conductance-based neuron with the addition of the refractory period. This is not surprising, since the noise term becomes negligible compared to mean inputs in the small *a* limit. In Fig. 9(b), we show that Eq. (B4) gives a good description of the transfer function predicted by the mean field theory in the suprathreshold regime.

#### b. Subthreshold regime *v*_max_ *> α* (*θ* > *μ*)

First, we consider *α* < *v*_min_ (*μ* < *V*_*r*_). For every value of *v*, the integral in *x* in Eq. (A13) has a maximum in the integration interval; hence, it can be performed through the saddle-point method and gives

(B5)
$$\frac{1}{\nu }-\tau_{rp}=\sqrt{\frac{4\pi \chi \tau }{a^{2}K\left(\alpha^{2}+1\right)}}\int_{v_{\text{min}}}^{v_{\text{max}}}dv\text{exp}\left[\frac{F(v)-F(\alpha )}{a}\right].$$

In the last equation, the exponent in the integrand has a minimum for *v* = *α* and is maximum at *v* = *v*_max_; we expand the exponent around *v* = *v*_max_ and, keeping term up to the first order, obtain

(B6)
$$\frac{1}{\nu }-\tau_{rp}=\tau \sqrt{\frac{\pi a^{2}K\tau }{\chi \left(\alpha^{2}+1\right)}}\frac{v_{\text{max}}^{2}+1}{\left|v_{\text{max}}-\alpha \right|}\text{exp}\left[\frac{F\left(v_{\text{max}}\right)-F(\alpha )}{a}\right].$$

In the regime *v*_min_ *< α* < *v*_max_, the integral in *v* of Eq. (A13) can be divided into three parts:

(B7)
$$\int_{v_{\text{min}}}^{v_{\text{max}}}dv=\int_{v_{\text{min}}}^{\alpha -\epsilon }dv+\int_{\alpha -\epsilon }^{\alpha +\epsilon }dv+\int_{\alpha +\epsilon }^{v_{\text{max}}}dv;$$

the third integral is analogous to the case *α* < *v*_min_, and, hence, it has an exponential dependency on the parameters and dominates the other terms. In Fig. 9(a), we show that Eq. (B6) gives a good description of the transfer function predicted by the mean field theory for *μ* < *θ*.

### 2. Single-neuron CV of ISI at strong coupling

In this section, we provide details of the derivation of the approximated expressions of the response CV. Starting from the mean field result of Eq. (A14), we compute integrals using the approach discussed above.

#### a. Suprathreshold regime *v*_max_ *< α* (*θ* < *μ*)

The inner integral in Eq. (A14) yields in the small *a* limit

(B8)
$$\int_{-\infty }^{z}dw\frac{1}{w^{2}+1}\text{exp}\left[\frac{F(z)-F(w)}{a}\right]=\frac{a}{z^{2}+1}\frac{1}{\frac{dF(z)}{dz}},$$

from which we obtain

(B9)
$${\text{CV}}^{2}=a\frac{\nu^{2}{\left(aK\tau \right)}^{3}}{a^{2}K^{2}\chi }[\text{log}\left(\frac{v_{\text{min}}-\alpha }{v_{\text{max}}-\alpha }\right)+\frac{-3\alpha^{2}+4\alpha v_{\text{max}}+1}{2{\left(\alpha -v_{\text{max}}\right)}^{2}}-\frac{-3\alpha^{2}+4\alpha v_{\text{min}}+1}{2{\left(\alpha -v_{\text{min}}\right)}^{2}}];$$

hence, the rescaling needed to preserve the deterministic component *a* ~ 1/*K* produces CV^2^ ~ *a* ≪ 1. We validate this result numerically in Figs. 9(h) and 11(f).

#### b. Subthreshold regime *v*_max_ *> α* (*θ* > *μ*)

The integral defining the CV [Eq. (A14)] can be expressed as

(B10)
$$\int_{-\infty }^{v}dz\text{ exp}\left[\frac{F(v)-F(z)}{a}\right]g(z)=\int_{-\infty }^{v^{*}}dz\text{ exp}\left[\frac{F(v)-F(z)}{a}\right]g(z)+\int_{v^{*}}^{v}dz\text{ exp}\left[\frac{F(v)-F(z)}{a}\right]g(z)$$

with

(B11)
$$g(z)={\left\{\int_{-\infty }^{z}dw\frac{1}{w^{2}+1}\text{exp}\left[\frac{F(z)-F(w)}{a}\right]\right\}}^{2}\;v^{*}=\alpha -\epsilon .$$

The first integral gives

(B12)
$$\int_{-\infty }^{v^{*}}dz\text{ exp}\left[\frac{F(v)-F(z)}{a}\right]g(z)=\frac{a^{3}}{{\left(v^{*}+1\right)}^{2}{\left[\frac{dF\left(v^{*}\right)}{dz}\right]}^{3}}.$$

In the second integral,

(B13)
$$g(z)=\frac{a\pi }{{\left(\alpha^{2}+1\right)}^{2}\frac{d^{2}F(\alpha )}{dz^{2}}}\text{exp}\left[\frac{2F(z)-2F(\alpha )}{a}\right],$$

from which we get

(B14)
$$\int_{v^{*}}^{v}dz\text{ exp}\left[\frac{F(v)+F(z)-2F(\alpha )}{a}\right]\frac{a\pi }{{\left(\alpha^{2}+1\right)}^{2}\frac{d^{2}F(\alpha )}{dz^{2}}}.$$

Integrating in *z*, we obtain

(B15)
$$\int_{v^{*}}^{v}dz\text{ exp}\left[\frac{F(z)}{a}\right]=\frac{a}{\frac{dF(v)}{dz}}\text{exp}\left[\frac{F(v)}{a}\right].$$

Integrating in *v*, we obtain

(B16)
$${\text{CV}}^{2}=\frac{8\chi^{2}\nu^{2}\pi }{{\left(\alpha^{2}+1\right)}^{2}\frac{d^{2}F(\alpha )}{dz^{2}}{\left(\frac{dF\left(v_{\text{max}}\right)}{dz}\right)}^{2}}{\left(\frac{\text{exp}\left[\frac{F\left(v_{\text{max}}\right)-F(\alpha )}{a}\right]}{\sqrt{a}K}\right)}^{2}.$$

Using Eq. (B6), we obtain

(B17)
$$\text{CV}=1-\nu \tau_{rp},$$

which corresponds to the CVof the ISI of a Poisson process with dead time, with rate *ν* and refractory period *τ*_*rp*_. We validate this result numerically in Figs. 9(d) and 11(c).

### 3. Scaling relations preserving firing in the strong-coupling limit

In this section, we use the simplified expressions derived above to define scaling relations of *a* with *K* which preserves neural response in the strong-coupling limit. Importantly, the scaling defined here depends on the operating regime of the neuron, i.e., on the asymptotic value of *μ*.

In the limit of large *K*, terms in Eq. (A8) can be written as

(B18)
$$\tau^{-1}=aK\nu_{X}(1+\eta g\gamma ),\;\omega^{-1}=\nu_{X}(1+\eta g\gamma ),\;\chi^{-1}=\nu_{X}\left(1+\eta g^{2}\gamma \right),$$

while *μ*, $E_{D}$, $E_{S}$, *v*_max_, *α*, and the function F(x) are independent of *K*, *a*, and *ν*_*E*_. We show in the previous section that the single-neuron transfer function is given by

(B19)
$$\frac{1}{\nu }=\tau_{rp}+\frac{Q}{\nu_{E}}$$

with

(B20)
$$Q=\{\begin{array}{ll} \left(\frac{1}{\sqrt{a}K}\text{exp}\frac{F\left(v_{\text{max}}\right)-F(\alpha )}{a}\right)\sqrt{\frac{\pi \left(1+\eta g^{2}\gamma \right)}{{\left(1+\eta g\gamma \right)}^{3}\left(\alpha^{2}+1\right)}}\frac{v_{\text{max}}^{2}+1}{v_{\text{max}}-\alpha |} & \text{for }\mu <\theta \\ \frac{1}{aK(1+\eta g\gamma )}\text{log}\left(\frac{\mu -\theta }{\mu -V_{r}}\right) & \text{for }\mu >\theta \end{array}$$

For *μ* > *θ*, the parameters *a* and *K* in Eq. (B20) appear only in the combination *aK*. It follows that a rescaling

(B21)
$$a\sim \frac{1}{K}$$

leaves invariant the neural response for large *K*. For *μ* < *θ*, Eq. (B20), and hence the transfer function, is invariant under the rescaling

(B22)
$$K\sim \frac{1}{\sqrt{a}}\text{exp}\left[\frac{F\left(v_{\text{max}}\right)-F(\alpha )}{a}\right].$$

In Figs. 11(a) and 11(d), we show neural responses computed for different values of *K* with *a* rescaled according to Eqs. (B21) or (B22); as predicted, the network transfer function remains invariant as *K* increases. Note that the response remains nonlinear in the limit of large *K*; we show in the next section that, in the network case, because of the self-consistency relation, nonlinearities are suppressed by the scaling relation.

Finally, from Figs. 11(c) and 11(f), we see that the rescaling preserves the CV for *μ* < *θ* and suppresses it for *μ* > *θ*. In the case *μ* < *θ*, the CV is given by Eq. (B17). This expression shows that the scaling relation of Eq. (B22) also leaves invariant the CV. Interestingly, in some parameter regime, the CV in Figs. 9(d) and 11(c) shows a nonmonotonic behavior with *ν*_*X*_ which is not captured by Eq. (B17). In particular, a CVabove one is observed when *μ* is below the reset *V*_*r*_. As pointed out in Ref. [88], this supra-Poissonian firing is explained by the fact that, when *μ* < *V*_*r*_, spiking probability is higher just after firing that it is afterward. In agreement with this interpretation, we find that the nonmonotonic behavior of the CV disappears in the large *K* limit, where the region of inputs for which *μ* < *V*_*r*_ becomes negligible. Thus, our analysis shows that the irregularity of firing is preserved in the strong-coupling limit of a single neuron with *μ* < *θ*.

In the case *μ* > *θ*, the CV is given by Eq. (B9). This expression shows that the scaling relation of Eq. (B21) produces a CV which decreases as 1/*K* in the strong-coupling limit. It follows that, in a single neuron with *μ* > *θ*, the strong-coupling limit produces finite firing that is regular.

Starting from the next section, we focus our attention to a network of conductance-based neurons. Since we are interested in describing the irregular firing observed in the cortex, we focus our study on networks with *μ* < *θ*.

## APPENDIX C: FIRING RATE AND SCALING RELATION IN LEAKY INTEGRATE-AND-FIRE NEURON MODELS WITH VOLTAGE-DEPENDENT CURRENTS

In the main text, we show that, when coupling is strong and *a* ≪ 1, the response of a single LIF neuron with conductance-based synapses is well approximated by Eq. (12), i.e., the Kramers escape rate. Using this expression, we show that the scaling relation of Eq. (14) allows finite firing in a single neuron and in networks of neurons. Here, we show that the first-order approximation of this scaling, i.e., *a* ~ 1/log(*K*), appears also in neuron models with additional biophysical details, such as spike-generating currents [44] and voltage-gated subthreshold currents [23], as long as coupling is strong, *a* is small, and synapses are conductance based.

We consider integrate-and-fire models featuring voltage-dependent currents, indicated here as *ϕ*(*V*), and conductance-based synapses. In these models, the membrane potential dynamics can be written as

(C1)
$$C_{j}\frac{dV_{j}}{dt}=-\underset{A=L,E,I}{\sum }g_{A}^{j}\left(V_{j}-E_{A}\right)+\psi (V).$$

In the LIF, *ψ*(*V*) = 0 and Eq. (C1) reduces to Eq. (1) analyzed in the main text. In the exponential integrate-and-fire model (EIF) [44], the function *ψ*(*V*) = Δ*Tg*_*L*_ exp[(*V* − *θ*)/Δ*T*] describes the spike generation current; in this model, once the membrane potential crosses the threshold *θ*, it diverges to infinity in finite time. The current generated by inward-rectifier voltage-gated channels, such as the one recently reported in Ref. [23], is captured by an expression of the form *ψ*(*V*) = −*g*_in_(*V*)(*V* − *E*_in_), where *g*_in_(*V*) and *E*_in_ represent the conductance and the reversal potential of the channels, respectively; in the case of Ref. [23], 1/*g*_in_(*V*) is shown to be well approximated by a linear increasing function of *V*.

The dynamics Eq. (C1), following an approach analogous to the one we use for the derivation of Eq. (4), can be approximated by

(C2)
$$\tau \frac{dV}{dt}=-\frac{\partial H(V)}{\partial V}+\sigma \sqrt{\tau }\zeta ,\;H(V)=\frac{1}{2}{\left(V-\mu \right)}^{2}-\frac{\tau }{\tau_{L}g_{L}}\int^{V}\psi (x)dx,$$

where *ζ* is a white noise term, with zero mean and unit variance density, while *τ*, *μ*, and *σ*(*V*) are as in Eq. (5). In what follows, as in the main text, we use the effective time constant approximation [40]—i.e., we neglect the multiplicative component of the noise term in Eq. (C2)—and make the substitution *σ*(*V*) → *σ*(*μ**), where *μ** is the mean value of the membrane potential dynamics.

The firing rate of a neuron following Eq. (C2) can be computed exactly using Eq. (A5) and is given by

(C3)
$$\nu ={\left[\tau_{rp}+\frac{2\tau }{\sigma^{2}}\int_{-\infty }^{\infty }dx\int_{\text{max}\left(V_{r},x\right)}^{\infty }\text{exp}\left(\frac{H(z)-H(x)}{\sigma^{2}}\right)dz\right]}^{-1}.$$

In what follows, we provide a more intuitive derivation of the single-neuron response, which is valid in the biologically relevant case of *a* ≪ 1. The function H in Eq. (C2) can be thought of as an energy function which drives the dynamics of the membrane potential. In the case of LIF neurons, H is a quadratic function with a minimum at *V* = *μ*. In neuron models with a spike generation current, such as the EIF model [44], the shape of the function H far from threshold is qualitatively similar to that of the LIF model (with a minimum at *V* = *μ**) but becomes markedly different close to threshold, where the potential energy has a maximum at *V* = *θ** and goes to −∞ for *V* > *θ**. Here, we focus on the case in which additional subthreshold voltage-gated currents do not lead to additional minima of the energy function, a scenario that can happen with potassium inward-rectifier currents (e.g., see Ref. [89], Chap. 4.4.3). In models in which H has a single minimum in the subthreshold range at *μ** and a maximum at *θ**, the firing rate of a neuron when input noise is small (i.e., when *a* ≪ 1) can again be computed using the Kramers escape rate, which gives the average time it takes for the membrane potential to go from *μ** to *θ** (see Ref. [43], Sec. V.5.3):

(C4)
$$\frac{1}{\nu }-\tau_{rp}=\frac{2\pi \bar{\tau }\bar{ϒ}}{aK\nu_{X}}\text{exp}\left(\frac{\bar{\Delta }}{a}\right),$$

where

(C5)
$$\bar{ϒ}={\left({{\frac{d^{2}H}{dV^{2}}|}_{\theta^{*}}\frac{d^{2}H}{dV^{2}}|}_{\mu^{*}}\right)}^{-1/2},\;\bar{\Delta }=\frac{H\left(\theta^{*}\right)-H\left(\mu^{*}\right)}{\bar{\sigma }},\;\bar{\tau }=aK\nu_{X}\tau ,\;\bar{\sigma }=\frac{\sigma }{\sqrt{a}},$$

while $\bar{.}$ indicates quantities that remain of the order of 1 in the small *a* limit, provided the external inputs *ν*_*X*_ are at least of the order of 1/(*aKτ*_*L*_). Equation (C4) is the generalization of Eq. (12) to the case of integrate-and-fire neuron models with voltage-dependent currents; it shows that, at the dominant order, finite firing emerges if *a* ~ 1/ log *K*. Moreover, Eq. (C4) shows that corrections to the logarithmic scaling depend on the specific type of voltage-dependent currents used in the model.

## APPENDIX D: CALCULATIONS IN THE STRONG-COUPLING REGIME—NETWORKS

In this section, we show how the results on the strong-coupling limit of single-neuron response can be generalized to the network case. First, we analyze the problem in the case in which excitatory and inhibitory neurons have the same biophysical properties (model *A*). In this model, we start by discussing the results using the effective time constant approximation and then discuss the full results. Then, we study the case in which excitatory and inhibitory neurons have different biophysical properties (model *B*).

### 1. Model *A*, effective time constant approximation

As discussed in the main text, the network response in model *A* with the effective time constant approximation is obtained by solving the self-consistency condition given by Eqs. (19) and (10). At strong coupling, this condition can be simplified to the form of Eq. (12). In the strong-coupling limit, when *ν*_*X*_ ≫ 1/*aKτ*_*L*_ and *ν* ≫ 1/*τ*_*rp*_, the right-hand side of Eq. (10) depends on *ν* and *ν*_*X*_ only through their ratio. Therefore, we look for solutions of the simplified self-consistency condition with a Taylor expansion

(D1)
$$\frac{\nu }{\nu_{X}}=\overset{k=\infty }{\underset{k=1}{\sum }}\rho_{k}x^{k-1},\;x=\tau_{rp}\nu_{X}.$$

Keeping only terms up to first order in *x*, the self-consistency condition becomes

$$\frac{1}{\rho_{1}}-\left(1+\frac{\rho_{2}}{\rho_{1}^{2}}\right)x=Q\left(\rho_{1}\right)+{\rho_{2}\frac{dQ(y)}{dy}|}_{y=\rho_{1}}x,$$

from which we find

(D2)
$$\rho_{1}=\frac{1}{Q\left(\rho_{1}\right)}$$

The solution of Eq. (D2) provides the linear component of the network response; this is preserved in the strong-coupling limit with an expression analogous to Eq. (14) but with

$$\frac{r_{E}}{\nu_{X}}=1+\rho_{1},\;\frac{r_{I}}{\nu_{X}}=\rho_{1}.$$

This uniquely defines a scaling between *a* and *K* [see Fig. 3(a) for an example of the scaling function]. We test the validity of our result in Fig. 3(b). The numerical analysis shows that, as *K* increases, the scaling relation prevents saturation and suppression of the network response. However, unlike what happens in the single-neuron case, the shape of the transfer function is not preserved and becomes increasingly linear as *K* becomes larger. This is analogous to what happens in the balanced state model [7,8,10,85], where the network transfer function becomes linear in the strong-coupling limit. For the case under investigation here, we can understand this suppression of nonlinearities by looking at the second-order terms in the expansion of Eq. (D1). Keeping the dominant contribution in *a*, we find

(D3)
$$\rho_{2}\sim a\frac{\rho_{1}{\bar{\sigma }}^{2}}{2{\bar{v}}_{\text{max}}\left(\bar{\sigma }\frac{d\mu }{dy}+(\theta -\mu )\frac{d\bar{\sigma }}{dy}\right)}.$$

Hence, *ρ*_2_ goes to zero as *a* decreases, producing a linear transfer function. This follows directly from the self-consistency relation and is not present in the single-neuron case, where, in fact, a nonlinear transfer function is observed in the large *K* limit. Figure 3(b) shows that linearity is reached really slowly with *K*; this follows directly from Eq. (D3), where the suppression of nonlinear terms is controlled by *a*, which slowly goes to zero with *K* (approximately logarithmically).

### 2. Model *A*, multiplicative noise

In this section, we generalize the approach used above, relaxing the effective time constant approximation. As discussed in Appendix B, Eq. (A13) in the strong-coupling limit becomes

(D4)
$$\frac{1}{\nu }=\tau_{rp}+\frac{Q}{\nu_{X}}$$

with

(D5)
$$Q=\frac{1}{\sqrt{a}K}\text{exp}\left[\frac{F\left(v_{\text{max}}\right)-F(\alpha )}{a}\right]\sqrt{\frac{\pi \left[1+\frac{\nu }{\nu_{X}}\left(1+g^{2}\gamma \right)\right]}{{\left[1+\frac{\nu }{\nu_{X}}(1+g\gamma )\right]}^{3}\left(\alpha^{2}+1\right)}}\frac{v_{\text{max}}^{2}+1}{\left|v_{\text{max}}-\alpha \right|}$$

and

(D6)
$$\tau^{-1}=aK\omega^{-1},\;\omega^{-1}=\nu_{X}\left[1+\frac{\nu }{\nu_{X}}(1+g\gamma )\right],\;\chi^{-1}=\nu_{X}\left[1+\frac{\nu }{\nu_{X}}\left(1+g^{2}\gamma \right)\right],\;\mu =\frac{E_{E}+\frac{\nu }{\nu_{X}}\left(E_{E}+g\gamma E_{I}\right)}{1+\frac{\nu }{\nu_{X}}(1+g\gamma )},\;E_{S}=\frac{E_{E}+\frac{\nu }{\nu_{X}}\left(E_{E}+g^{2}\gamma E_{I}\right)}{1+\frac{\nu }{\nu_{X}}\left(1+g^{2}\gamma \right)},\;E_{D}=\frac{\left(E_{E}-E_{I}\right)\sqrt{\left(1+\frac{\nu }{\nu_{X}}\right)\frac{\nu }{\nu_{X}}g^{2}\gamma }}{1+\frac{\nu }{\nu_{X}}\left(1+g^{2}\gamma \right)}.$$

Here, we assume *aK* ≫ 1/*τ*_*L*_*ν*_*X*_ so that the function Q depends on *ν* and *ν*_*X*_ only through the combination *ν*/*ν*_*X*_. We show below that a scaling relation analogous to that of single neurons holds; hence, for *K* large enough *aK* ≫ 1/*τ*_*L*_*ν*_*X*_ is automatically implemented. To solve the self-consistency condition, we express the firing rate *ν* with a Taylor expansion

(D7)
$$\tau_{rp}\nu =\overset{k=\infty }{\underset{k=1}{\sum }}\rho_{k}x^{k},\;x=\tau_{rp}\nu_{X}.$$

Note that in Eq. (D7) we assume *ρ*_0_ = 0; we come back to this point at the end of the section. Under this assumption, $y≔\nu /\nu_{X}=\sum_{k=1}^{k=\infty }\rho_{k}x^{k-1}$ and the function Q depends only on powers of the dimensionless variable *x*. Keeping only terms up to first order in *x*, Eq. (D4) becomes

(D8)
$$\frac{1}{\rho_{1}}-\left(1+\frac{\rho_{2}}{\rho_{1}^{2}}\right)x=Q\left(\rho_{1}\right)+{\rho_{2}\frac{dQ(y)}{dy}|}_{y=\rho_{1}}x,$$

from which we find

(D9)
$$\rho_{1}=\frac{1}{Q\left(\rho_{1}\right)}.$$

The solution of Eq. (D9) provides the linear component of the network response, i.e., its gain; we discuss this function in more detail at the end of this section.

From Eq. (D9), we find that the network gain *ρ*_1_ is preserved in the strong-coupling limit if the factor

(D10)
$$\frac{1}{\sqrt{a}K}\text{exp}\left[\frac{F\left(v_{\text{max}}\right)-F(\alpha )}{a}\right]$$

is constant. Equation (D10) uniquely defines a scaling between *a* and *K* [see Fig. 12(c) for an example of the scaling function]. We test the validity of the scaling in Fig. 12 as follows: Given a set of parameters *a*, *K*, and *ρ*_1_, we compute numerically the transfer function from Eq. (A13); then we increase *K*, determine the corresponding change in *a* using Eq. (D10), and compute again the transfer function—results of this procedure are shown in Fig. 12(a). The numerical analysis shows that, as *K* increases, our scaling relation prevents saturation and the network response remains finite.

As in the case with diffusion approximation, the shape of the transfer function is not preserved by the scaling and an increasing linear response is observed. We can understand this suppression of nonlinearities by looking at the second-order terms in the expansion of Eq. (D4); we find

(D11)
$$\rho_{2}=\frac{-\rho_{1}^{2}}{\rho_{1}\frac{d\text{log}[Q(y)]}{dy}+1},$$

and, keeping the dominant contribution in 1/*a* at the denominator,

(D12)
$$\rho_{2}\sim \frac{-a\rho_{1}}{{\frac{dF\left[v_{\text{max}}(y),y\right]}{dy}|}_{\rho_{1}}+{\frac{dF[\alpha (y),y]}{dy}|}_{\rho_{1}}}.$$

Hence, *ρ*_2_ goes to zero as *a* decreases, producing a linear transfer function. The nonlinearities at low rate in Fig. 12(a) (e.g., see red and yellow lines) show that our assumption *ρ*_0_ = 0 is not valid, in general. However, it turns out that the above-defined scaling relation suppresses also these nonlinearities in the limit of strong coupling (e.g., blue and cyan lines).

We now characterize the dependency of the transfer function gain, i.e., its slope, on network parameters. For fixed network parameters, the network gain *ρ*_1_ is defined as the solution of Eq. (D9); solutions as a function of *a* and *g* are shown in Fig. 12(e). At fixed values of *a*, the gain initially decreases as *g* increases, and, for *g* large enough, the opposite trend appears. This behavior is due to two different effects which are produced by the increase of *g*: On one hand, it increases the strength of recurrent inhibition; on the other hand, it decreases the equilibrium membrane potential *μ* and brings it closer to the inhibitory reversal potential *E*_*i*_, which, in turn, weakens inhibition [see Fig. 12(f)]. Figure 12(e) shows that the gain is finite only for a finite range of the parameter *g*; divergences appear because recurrent inhibition is not sufficiently strong to balance excitation. At small *g*, the unbalance is produced by weak efficacy of inhibitory synapses; at large *g*, inhibition is suppressed by the approach of the membrane potential to the reversal point of inhibitory synapses. Increasing the value of *a* produces an upward shift in the curve and, at the same time, decreases the range of values in which the gain is finite. The observed decrease in gain generated at low values of *g* is observed also in networks of current-based neurons [10], where the gain is found to be 1/(*gγ* – 1). Finally, we note that the difference between conductance- and current-based model decreases with *a*.

To conclude this analysis, we give an approximated expression of the probability distribution of the membrane potential of Eq. (A12) which, in the strong-coupling limit, becomes

(D13)
$$P(V)=\frac{\nu \omega }{\left|v_{\text{max}}-\mu \right|}\left[\frac{u{\left(V_{\text{max}}\right)}^{2}+1}{u{\left(V\right)}^{2}+1}\right]\frac{e^{\left[F\left(v_{\text{max}}\right)-F(V)\right]/a}}{aK},$$

where *V*_max_ is the value of the membrane potential *V* which maximizes the integrand of Eq. (A12) while the function *u*() is defined in Eq. (A15). Examples of the probability distribution and the corresponding approximated expressions are given in Fig. 12(d).

### 3. Model *B*, multiplicative noise

In this section, we generalize the results obtained so far to the case of networks with excitatory and inhibitory neurons with different biophysical properties.

#### a. Model definition

Here, we take into account the diversity of the two types of neurons with

(D14)
$$\tau_{j}=\tau_{E},\;a_{jm}=a_{EX},a_{EE},a_{EI},$$

for excitatory neurons and

(D15)
$$\tau_{j}=\tau_{I},\;a_{jm}=a_{IX},a_{IE},a_{EE},$$

for inhibitory neurons. We use the parametrization

(D16)
$$a_{EX}=a_{E},\;a_{EE}=a_{E},\;a_{EI}=g_{E}a_{E},\;a_{IX}=a_{I},\;a_{IE}=a_{I},\;a_{II}=g_{I}a_{I},$$

and

(D17)
$$K_{EX}=K_{E},\;K_{EE}=K_{E},\;K_{EI}=\gamma_{E}K_{E},\;K_{IX}=K_{I},\;K_{IE}=K_{I},\;K_{II}=\gamma_{I}K_{I}.$$

Equation (1) becomes

(D18)
$$\tau_{E}\frac{dV_{E}}{dt}=-\left(V_{E}-\mu_{E}\right)-\sigma_{E}\left(V_{E}\right)\sqrt{\tau_{E}}\zeta_{E},\;\tau_{I}\frac{dV_{I}}{dt}=-\left(V_{I}-\mu_{I}\right)-\sigma_{I}\left(V_{I}\right)\sqrt{\tau_{I}}\zeta_{I}.$$

The expressions for excitatory neurons are

(D19)
$$\tau_{E}^{-1}=\tau_{L,E}^{-1}+a_{E}K_{E}\omega_{E}^{-1},\;\omega_{E}^{-1}=\nu_{EX}+\nu_{E}+g_{E}\gamma_{E}\nu_{I},\;\mu_{E}=\tau_{E}\left\{E_{L}+a_{E}K_{E}\tau_{L,E}\left[\nu_{EX}E_{E}+\nu_{E}E_{E}+\nu_{I}g_{E}\gamma_{E}E_{I}\right]\right\}\;\sigma_{E}^{2}=a_{E}^{2}K_{E}\frac{\tau_{E}}{\chi_{E}}\left[{\left(V-E_{S,E}\right)}^{2}+E_{D,E}^{2}\right],\;\chi_{E}^{-1}=\nu_{EX}+\nu_{E}+g_{E}^{2}\gamma_{E}\nu_{I},\;E_{S,E}=\chi_{E}\left[\nu_{EX}E_{E}+\nu_{E}E_{E}+\nu_{I}g_{E}^{2}\gamma_{E}E_{I}\right)],\;E_{D,E}=\chi_{E}\sqrt{\left(\nu_{EX}+\nu_{E}\right)g_{E}^{2}\gamma_{E}\nu_{I}}\left(E_{E}-E_{I}\right);$$

analogous expressions are valid for inhibitory neurons.

The firing rate is given by solving a system of two equations:

(D20)
$$\frac{1}{\nu_{E}}-\tau_{rp}=\frac{2\chi_{E}}{a_{E}^{2}K_{E}}\int_{v_{\text{min},E}}^{v_{\text{max},E}}dv\int_{-\infty }^{v}dx\frac{1}{x^{2}+1}\text{exp}\left[\frac{F_{E}(v)-F_{E}(x)}{a_{E}}\right],\;\frac{1}{\nu_{I}}-\tau_{rp}=\frac{2\chi_{I}}{a_{I}^{2}K_{I}}\int_{v_{\text{min},I}}^{v_{\text{max},I}}dv\int_{-\infty }^{v}dx\frac{1}{x^{2}+1}\text{exp}\left[\frac{F_{I}(v)-F_{I}(x)}{a_{I}}\right],$$

with

(D21)
$$F_{E}(x)=\frac{2\chi_{E}}{a_{E}K_{E}\tau_{E}}\left[\frac{1}{2}\text{log}\left(x^{2}+1\right)-\alpha_{E}\text{arctan}(x)\right],\;v_{\text{min},E}=\frac{V_{r}-E_{S,E}}{E_{D,E}},\;v_{\text{max},E}=\frac{\theta -E_{S,E}}{E_{D,E}},\;\alpha_{E}=\frac{\mu_{E}-E_{S,E}}{E_{D,E}},$$

and analogous expressions for the inhibitory population. The probability distribution of the membrane potential and the CV are straightforward generalizations of Eqs. (A12) and (A14).

#### b. Scaling analysis

We parametrize inputs to the two populations as *ν*_*EX*_ and *ν*_*IX*_ = *ην*_*EX*_. Using an analysis analogous to the one depicted above, we obtain a simplified expression for the self-consistency Eq. (D20) that is

(D22)
$$\frac{1}{\nu_{E}}-\tau_{rp}=\frac{Q_{E}\left(\nu_{E}/\nu_{EX},\nu_{I}/\nu_{EX}\right)}{\nu_{EX}},\;\frac{1}{\nu_{I}}-\tau_{rp}=\frac{Q_{i}\left(\nu_{E}/\nu_{EX},\nu_{I}/\nu_{EX}\right)}{\nu_{EX}},$$

where

(D23)
$$Q_{E}=\left[\frac{1}{\sqrt{a_{E}}K_{E}}\text{exp}\frac{F_{E}\left(v_{\text{max},E}\right)-F_{E}\left(\alpha_{E}\right)}{a_{E}}\right]\sqrt{\frac{\pi \left[1+\frac{\nu_{E}}{\nu_{EX}}+g_{E}^{2}\gamma_{E}\frac{\nu_{I}}{\nu_{EX}}\right]}{{\left[1+\frac{\nu_{E}}{\nu_{EX}}+g_{E}\gamma_{E}\frac{\nu_{I}}{\nu_{EX}}\right]}^{3}\left(\alpha_{E}^{2}+1\right)}\frac{v_{\text{max},E}^{2}+1}{\left|v_{\text{max},E}-\alpha_{E}\right|}}$$

and

(D24)
$$Q_{I}=\left[\frac{1}{\sqrt{a_{I}}K_{I}}\text{exp}\frac{F_{I}\left(v_{\text{max},I}\right)-F_{I}\left(\alpha_{I}\right)}{a_{I}}\right]\sqrt{\frac{\pi \left[\eta +\frac{\nu_{E}}{\nu_{EX}}+g_{I}^{2}\gamma_{I}\frac{\nu_{I}}{\nu_{EX}}\right]}{{\left[\eta +\frac{\nu_{E}}{\nu_{EX}}+g_{I}\gamma_{I}\frac{\nu_{I}}{\nu_{EX}}\right]}^{3}\left(\alpha_{I}^{2}+1\right)}\frac{v_{\text{max},I}^{2}+1}{\left|v_{\text{max},I}-\alpha_{I}\right|}}.$$

We investigate the solution in the strong-coupling limit using an expansion:

(D25)
$$\tau_{rp}\nu_{E}=\overset{k=\infty }{\underset{k=1}{\sum }}\rho_{k}^{E}x^{k},\;\tau_{rp}\nu_{I}=\overset{k=\infty }{\underset{k=1}{\sum }}\rho_{k}^{I}x^{k},\;x=\tau_{rp}\nu_{EX},$$

and obtain

(D26)
$$\rho_{1}^{E}=\frac{1}{Q_{E}\left(\rho_{1}^{E},\rho_{1}^{I}\right)},\;\rho_{1}^{I}=\frac{1}{Q_{I}\left(\rho_{1}^{E},\rho_{1}^{I}\right)}.$$

Equation (D26) defines the gain of the excitatory and inhibitory populations. As for model *A*, requiring that network gain is preserved in the large *K* limit is equivalent to assuming the products

(D27)
$$\frac{1}{\sqrt{a_{j}}K_{j}}\text{exp}\frac{F_{j}\left(v_{\text{max},j}\right)-F_{j}\left(\alpha_{j}\right)}{a_{j}}$$

constant; these constraints defines how synaptic strength should scale with *K* to preserve the response gain. We note that, since $F_{j}\left(v_{\text{max},j}\right)-F_{j}\left(\alpha_{j}\right)$ is different for the two populations, in the general case there are two different scalings for the two populations; in Fig. 13, we verify this prediction.

## APPENDIX E: SIMULATIONS VS THEORY

All the results shown in the main text are based on the mean field analysis of the network dynamics. In this section, we investigate how the predictions of the mean field theory compare to numerical simulations of networks of conductance-based neurons.

Using the simulator Brian2 [71], we simulate the dynamics of networks of spiking neurons defined by Eq. (1). We investigate networks of *N*_*E*_ excitatory and *N*_*I*_ inhibitory neurons; the two groups are driven by two populations of Poisson units of size *N*_*EX*_ and *N*_*IX*_, respectively. Simulations are performed for *N*_*E*_ = *N*_*I*_ = *N*_*EX*_ = *N*_*IX*_ = 10*K* and 100*K*, with no significant differences between the two. We use uniformly distributed delays of excitatory and inhibitory synapses. Delays are drawn randomly and independently at each existing synapse from uniform distributions in the range [0, 10] ms (*E* synapses) and [0, 1] ms (*I* synapses). For fixed network parameters, the dynamics is simulated for 10 s with a time step of 10 *μ*s. We perform simulations for different values of *K*; the value of *a* is rescaled according to the scaling relation of Eq. (D10). From the resulting activity, we measure the firing rate, CV, and probability distribution of the membrane potential; results are shown in Fig. 14. Mean field predictions are in qualitative agreement with numerical simulations, and the agreement improves as *a* decreases. Deviations from mean field are expected to arise potentially from three factors: (i) finite size of conductance jumps due to presynaptic action potentials; (ii) correlations in synaptic inputs to different neurons in the network due to recurrent connectivity; (iii) temporal correlations in synaptic inputs due to non-Poissonian firing behavior. In our simulations, deviations due to (i) and (ii) become small when both *a* and the connection probability are small. Deviations due to (iii) become small when *ν* ≪ 1/*τ*_*rp*_, since, as shown in Eq. (B17) in Appendix B, the statistics of presynaptic neurons firing tend to those of a Poisson process. As predicted by the mean field analysis, with increasing *K* (and decreasing *a*) the network response becomes linear and approaches the asymptotic scaling; the firing remains irregular, as shown by the CV, and the membrane potential becomes Gaussian distributed.

## APPENDIX F: EFFECTS OF HETEROGENEITY IN THE CONNECTIVITY BETWEEN NEURONS

In this section, we describe how fluctuations in single cell properties modify the expressions described above; in particular, we investigate the effect of heterogeneities in the number of connections per neuron in the simplified framework of model *A*. The formalism described here is a generalization to networks of conductance-based neurons of the analysis done in Refs. [55,90] for networks of current-based neurons.

We assume that the *i*th neuron in the network receives projections from $K_{X}^{i}$, $K_{E}^{i}$, and $K_{I}^{i}$ external, excitatory, and inhibitory neurons, respectively. These numbers are drawn randomly from Gaussian distributions with mean *K* (*γK*) and variance Δ*K*^2^ (*γ*^2^Δ*K*^2^) for excitatory (inhibitory) synapses. Note that Δ*K*^2^ is assumed to be sufficiently small so that the probability to generate a negative number can be neglected. Fluctuations in the number of connections are expected to produce a distribution of rates in the population, characterized by mean and variance *ν* and Δ*ν*^2^. As a result, the rates of incoming excitatory and inhibitory spikes differ from cell to cell and become

(F1)
$$K_{E}^{i}r_{E}^{i}=K\left(r_{E}+\Delta_{E}z_{E}^{i}\right),\;K_{I}^{i}r_{I}^{i}=\gamma K\left(r_{I}+\Delta_{I}z_{I}^{i}\right),\;r_{E}=\nu +\nu_{X},\;r_{I}=\nu ,\;\Delta_{E}^{2}={\text{CV}}_{K}^{2}\left(\nu^{2}+\nu_{X}^{2}\right)+\frac{\Delta \nu^{2}}{K}\approx {\text{CV}}_{K}^{2}\left(\nu^{2}+\nu_{X}^{2}\right),\;\Delta_{I}^{2}={\text{CV}}_{K}^{2}\nu^{2}+\frac{\Delta \nu^{2}}{\gamma K}\approx {\text{CV}}_{K}^{2}\nu^{2},$$

where *r*_*E,I*_ are the average presynaptic rates and $z_{E,I}^{i}$ are realizations of a quenched normal noise with zero mean and unit variance, fixed in a given realization of the network connectivity. Starting from Eq. (F1), the rate *ν*^*i*^ of the cell is derived as in the case without heterogeneities; the main difference is that it is now a function of the particular realizations of $z_{E}^{i}$ and $z_{I}^{i}$. The quantities *ν* and Δ*ν*^2^ are obtained from population averages through the self-consistency relations

(F2)
$$\nu =\langle \nu \left(z_{E},z_{I}\right)\rangle ,\;\Delta \nu^{2}=\langle \nu {\left(z_{E},z_{I}\right)}^{2}\rangle -\nu^{2},$$

where 〈·〉 represents the Gaussian average over the variables *z*_*E*_ and *z*_*I*_. Once *ν* and Δ*ν*^2^ are known, the probability distribution of firing rate in the population is given by

(F3)
$$P(\nu )=\frac{1}{2\pi }\int_{-\infty }^{\infty }dz_{E}dz_{I}e^{-z_{E}^{2}/2}e^{-z_{I}^{2}/2}\delta \left[\nu -\nu \left(z_{E},z_{I}\right)\right].$$

As shown in the main text [Fig. 4(a)], Eq. (F3) captures quantitatively the heterogeneity in rates observed in numerical simulations.

In the large *K* (small *a*) limit, the mathematical expressions derived above simplify significantly. First, as long as the parameter *μ*^*i*^ of the *i*th neuron is below threshold, its rate is given by an expression analogous to Eq. (12) which, for small Δ_*E,I*_, can be written

(F4)
$$Q_{i}=Q\text{exp}\left(\Gamma z_{i}\right),\;\Gamma^{2}={\left(\frac{\partial v_{\text{max}}^{2}}{\partial r_{E}}\Delta_{E}\right)}^{2}+{\left(\frac{\partial v_{\text{max}}^{2}}{\partial r_{I}}\Delta_{I}\right)}^{2},$$

where *z*^*i*^ is generated from a Gaussian random variable with zero mean and unit variance. Moreover, if responses are far from saturation, the single rate can be written as

(F5)
$$\nu_{i}=\frac{\nu_{X}}{Q_{i}}=\nu_{0}\text{exp}\left(-\Gamma z_{i}\right),\;\Gamma^{2}=\Omega^{2}\frac{{\text{CV}}_{K}^{2}}{a^{2}},\;\Omega^{2}=\left[{\left(a\frac{\partial v_{\text{max}}^{2}}{\partial \left(r_{E}/\nu_{X}\right)}\right)}^{2}{\left(\rho^{2}+1\right)}^{2}+{\left(a\frac{\partial v_{\text{max}}^{2}}{\partial \left(r_{I}/\nu_{X}\right)}\right)}^{2}\rho^{2}\right],$$

where *ν*_0_ is the rate in the absence of quenched noise [i.e., Eq. (20) in the main text]. It is easy to show that, in Eq. (F5), Ω^2^ is independent of *a*, *K*, and *ν*_*X*_ in the large *K* (small *a*) limit. Finally, as noted in Ref. [55], if the single-neuron rate can be expressed as an exponential function of a quenched variable *z*, Eq. (F3) can be integrated exactly and the distribution of rates is log-normal and given by

(F6)
$$P(\nu )=\frac{1}{\sqrt{2\pi }\Gamma \nu }\text{exp}\left(-\frac{{\left[\text{log}(\nu )-\text{log}\left(\nu_{0}\right)\right]}^{2}}{2\Gamma^{2}}\right).$$

Therefore, when the derivation of Eq. (F5) is valid, rates in the network should follow a log-normal distribution, with parameters given by

(F7)
$$\nu =\nu_{0}\text{exp}\left(\frac{\Gamma^{2}}{2}\right),\;\Delta \nu^{2}=\nu^{2}\left[\text{exp}\left(\frac{\Gamma^{2}}{2}\right)-1\right].$$

For Γ^2^ ≪ 1, we find Δ*ν*/*ν* ≈ Γ/2, which scales linearly with CV_*K*_, consistent with numerical results shown in Fig. 4(c).

## APPENDIX G: FINITE SYNAPTIC TIME CONSTANTS

In this section, we discuss the effect of the synaptic time constant on single-neuron and network responses. First, we derive an approximated expression for the single-neuron membrane time constant; we then compute approximated expressions which are valid for different values of the ratio *τ*_*S*_/*τ*; at the end of the section, we discuss the response of networks of neurons with large *τ*_*S*_/*τ*.

The single-neuron membrane potential dynamics is given by

(G1)
$$C_{j}{\dot{V}}_{j}(t)=-g_{L}^{j}\left(V_{j}-E_{L}\right)-\underset{A=E,I}{\sum }g_{A}^{j}(t)\left(V_{j}-E_{A}\right),\;\tau_{E}{\dot{g}}_{E}^{j}=-g_{E}^{j}+g_{L}^{j}\tau_{E}\underset{m}{\sum }a_{jm}\underset{n}{\sum }\delta \left(t-t_{m}^{n}-D\right),\;\tau_{I}{\dot{g}}_{I}^{j}=-g_{I}^{j}+g_{L}^{j}\tau_{I}\underset{m}{\sum }a_{jm}\underset{n}{\sum }\delta \left(t-t_{m}^{n}-D\right).$$

Using the effective time constant approximation [40], we have

(G2)
$$C\dot{V}=-g_{0}(V-\mu )-g_{EF}\left(\mu -E_{E}\right)-g_{IF}\left(\mu -E_{I}\right),\;\tau_{E}{\dot{g}}_{EF}=-g_{EF}+g_{L}\sigma_{E}\sqrt{\tau_{E}}\zeta_{E},\;\tau_{I}{\dot{g}}_{IF}=-g_{IF}+g_{L}\sigma_{I}\sqrt{\tau_{I}}\zeta_{I},$$

where *g*_*AF*_ represents the fluctuating component of the conductance *g*_*A*_, i.e.,

(G3)
$$g_{A}(t)=g_{A0}+g_{AF}(t)$$

and

(G4)
$$\langle \zeta_{A}(t)\zeta_{B}(t^{\prime })\rangle =\delta_{A,B}\delta \left(t-t^{\prime }\right),\;g_{0}=g_{L}+g_{E0}+g_{I0},\;g_{A0}=g_{L}a_{A}\tau_{A}R_{A},\;\sigma_{A}^{2}=a_{A}^{2}\tau_{A}R_{A}.$$

We are interested in stationary response, so we introduce the term

(G5)
$$z=\left(\mu -E_{E}\right)g_{EF}+\left(\mu -E_{I}\right)g_{IF}$$

with derivative

(G6)
$$\dot{z}=\left(\mu -E_{E}\right)\frac{-g_{EF}+g_{L}\sigma_{E}\sqrt{\tau_{E}}\zeta_{E}}{\tau_{E}}+\left(\mu -E_{I}\right)\frac{-g_{IF}+g_{L}\sigma_{I}\sqrt{\tau_{I}}\zeta_{I}}{\tau_{I}}.$$

Since we are interested in understanding the effect of an additional timescale, we can simplify the analysis assuming a unique synaptic timescale *τ*_*E*_ = *τ*_*I*_ = *τ*_*S*_ and obtain

(G7)
$$\tau_{S}\dot{z}=-z+\sigma_{z}\sqrt{\tau_{S}}\zeta ,\;\sigma_{z}^{2}=g_{L}^{2}\left[\sigma_{E}^{2}{\left(\mu -E_{E}\right)}^{2}+\sigma_{I}^{2}{\left(\mu -E_{I}\right)}^{2}\right].$$

To have the correct limit for *τ*_*S*_ → 0, we impose *a*_*A*_ = *a*_*A*0_*τ*_*L*_/*τ*_*S*_, where *a*_*A*0_ is the value of the synaptic efficacy in the limit of the instantaneous synaptic timescale. With these assumptions, the system equation becomes

(G8)
$$\tau \frac{dV}{dt}=-(V-\mu )-\sigma \sqrt{\frac{\tau }{\tau_{S}}}z,\;\tau_{s}\frac{dz}{dt}=-z+\sqrt{\tau_{S}}\zeta .$$

One can check that, in the limit *τ*_*S*_ → 0, the become analogous to those of the main text with $\eta =z/\sqrt{\tau_{S}}$. In what follows, we provide approximated expressions for the single-neuron transfer function in three regimes: small time constant [67,68], large time constant [70], and intermediate values [72]. We also note that a numerical procedure to compute the firing rate exactly for any value synaptic time constant was introduced recently, using Fredholm theory [91].

### 1. Single-neuron transfer function for different values of *τ*_*S*_/*τ*

For *τ*_*S*_/*τ* ≪ 1, as shown in Ref. [67,68], the firing rate can be computed with a perturbative expansion and is given by

(G9)
$$\frac{1}{\nu }=\tau \sqrt{\pi }\int_{{\tilde{v}}_{\text{min}}}^{{\tilde{v}}_{\text{max}}}dx[1+\text{erf}(x)],\;\tilde{v}(x)=\frac{x-\mu }{\sigma }-\tilde{\alpha }\sqrt{\frac{\tau_{S}}{\tau }}.$$

with $\bar{\alpha }=-\zeta (1/2)\approx 1.46$. As shown in Fig. 15, Eq. (G9) generates small corrections around the prediction obtained with instantaneous synapses and captures well the response for values *τ*_*S*_/*τ* ≲ 0.1.

For *τ*_*S*_/*τ* ≈ 1, as shown in Ref. [72] using the Rice formula [92], the single-neuron firing rate is well approximated by the rate of upward threshold crossing of the membrane potential dynamics without reset. Starting from Eq. (G8) and using the results of Ref. [72], we obtain

(G10)
$$\nu =\frac{1}{2\pi \sqrt{\tau \tau_{S}}}\text{exp}\left[-v_{\text{max}}^{2}\left(1+\frac{\tau_{S}}{\tau }\right)\right].$$

For *τ*_*S*_/*τ* ≫ 1, as shown in Ref. [70], the neuron fires only when fluctuations of *z* are large enough for *V* to be above threshold; the corresponding rate is given by

(G11)
$$\nu =\int_{v_{\text{max}}/\epsilon }^{\infty }dw\frac{e^{-w^{2}}}{\sqrt{\pi }}\frac{1}{\tau_{rp}+\tau \text{log}\left(\frac{v_{\text{min}}-\epsilon w}{v_{\text{max}}-\epsilon w}\right)},\;\epsilon =\sqrt{\frac{\tau }{\tau_{S}}}.$$

As shown in Fig. 15, Eq. (G11) captures the response for values *τ*_*S*_/*τ* ≳ 1 and predicts a strong suppression of response at larger *τ*_*S*_/*τ*.

Higher-order terms in the *τ*_*S*_/*τ* expansion could be computed using the approach described in Ref. [91]. However, Fig. 15 shows that Eqs. (G9)–(G11) are sufficient to capture quantitatively responses observed in numerical simulations for different regimes of *τ*_*S*_/*τ*. Equations (G9)–(G11) show that the single-neuron response is a nonlinear function of input rates; this nonlinearity prevents a scaling relation between *a* and *K* to rescue the suppression observed in Figs. 15 and 6(a).

### 2. Network response for *τ*_*S*_/*τ* larger than one

In this section, we study responses in networks of neurons with large *τ*_*S*_/*τ*. As in the case of instantaneous synapses, the network response can be obtained by solving the self-consistency relation given by the single-neuron transfer function using input rates

$$r_{E}=\nu_{X}+\nu ,\;r_{I}=\nu .$$

In particular, solutions of the implicit equation generated by Eq. (G11) give the network response in the region of inputs for which *τ*_*S*_/*τ* ≫ 1. In this region of inputs, assuming coupling to be strong, the implicit equation becomes

(G12)
$$\nu =\frac{\sqrt{\tau /\tau_{S}}}{\tau_{rp}v_{\text{max}}\sqrt{\pi }}\text{exp}\left(-v_{\text{max}}^{2}\frac{\tau_{S}}{\tau }\right).$$

Equation (G12), which is validated numerically in Fig. 16, implies that firing is preserved if $v_{\text{max}}\sqrt{\tau_{S}/\tau }$ is of the order of one, i.e., if

(G13)
$$\mu \sim \theta -\sigma \sqrt{\frac{\tau }{\tau_{S}}}\sim \theta -\frac{1}{\sqrt{K}}\frac{\sigma /\sqrt{a}}{\sqrt{\tau_{S}\left[\nu_{X}+\nu (1+g\gamma )\right]}}.$$

Combining the above equation with the definition of *μ*, we obtain Eq. (21), which captures the behavior of network response observed in numerical simulations for *τ*_*S*_/*τ* ≫ 1 (Figs. 6 and 16).

Equation (G12) can be used to understand the effect of connection heterogeneity in networks with large *τ*_*S*_/*τ*. In particular, generalizing the analysis of Appendix F, we find that rates in the network, in the limit of small CV_*K*_ and large *K*, are given by

(G14)
$$\nu_{i}=\nu_{0}\text{exp}\left[\Omega_{S}\frac{{\text{CV}}_{K}}{\sqrt{K}}z_{i}\right],$$

where *ν*_0_ is the population average in the absence of heterogeneity [i.e., the solution of Eq. (G12)] and *z*_*i*_ is a Gaussian random variable of zero mean and unit variance. The prefactor Ω_*S*_, which is independent of *a* and *K*, is given by

(G15)
$$\Omega_{S}^{2}=\left[{\left(\frac{\partial f\left(r_{E},r_{I}\right)}{\partial r_{E}}\right)}^{2}\left(\nu^{2}+\nu_{X}^{2}\right)+{\left(\frac{\partial f\left(r_{E},r_{I}\right)}{\partial r_{I}}\right)}^{2}\nu^{2}\right],\;f\left(r_{E},r_{I}\right)=\frac{v_{\text{max}}^{2}\tau_{S}}{K\tau }.$$

Equation (G15) is a generalization of Eq. (22) to the case of large *τ*_*S*_/*τ*. It shows that, in this limit, the state of the network is preserved with connection fluctuations up to ${\text{CV}}_{K}\sim 1/\sqrt{K}$.

## APPENDIX H: SHORT-TERM PLASTICITY

In the main text, we show that the finite synaptic time constant generates suppression of single-neuron response for large inputs. In this section, we investigate how this suppression is modified when short-term plasticity is taken into account. We focus our analysis on short-term depression, since this type of plasticity is the most commonly observed in cortical neurons when the presynaptic firing rate is large [93].

We consider a neuron receiving *K* (*γK*) excitatory (inhibitory) inputs, each with synaptic time constant *τ*_*S*_, from cells firing with Poisson statistics with a rate *r*_*E*_ = *ν*_*X*_, *r*_*I*_ = *ην*_*X*_. Following the approach of Refs. [94,95], we include synaptic depression in Eq. (G1) by assuming that a spike of the *m*th presynaptic neuron generates an input to the *j*th postsynaptic neuron given by *g*_*L*_*τ*_*S*_*a*_*jm*_*x*_*jm*_(*t*), where *x*_*jm*_(*t*) ∈ [0, 1] is the depression variable representing the fraction of available neurotransmitter at the synapse. We assume that *x*_*jm*_(*t*) evolves in time as

(H1)
$$\frac{dx_{jm}}{dt}=\frac{1-x_{jm}}{\tau_{D}},$$

in the absence of presynaptic spikes, and as

(H2)
$$x_{jm}\to x_{jm}(1-U),$$

in response to a presynaptic spike. In the model, *U* ∈ [0, 1] describes the fraction of available resources used to produce the postsynaptic input, while *τ*_*D*_ indicates the timescale over which such resources are regenerated. As shown in Refs. [94,95], *x*_*jm*_ satisfies the recursive relation

(H3)
$$x_{jm}\left(t_{m}^{n+1}\right)=1+\left[x_{jm}\left(t_{m}^{n}\right)(1-U)-1\right]e^{-\left(t_{m}^{n+1}-t_{m}^{n}\right)/\tau_{D}},$$

where $x_{jm}\left(t_{m}^{n+1}\right)$ and $x_{jm}\left(t_{m}^{n}\right)$ indicate the values of *x*_*jm*_ after *n* + 1th and *n*th presynaptic spike, respectively. With such synaptic dynamics, the statistics of inputs to single neurons are given by Eq. (G8) with the following substitutions:

(H4)
$$\tau^{-1}\to \tau_{x}^{-1}=\tau_{L}^{-1}+aK\left(x_{E}r_{E}+x_{I}r_{I}g\gamma \right),\;\mu \to \mu_{x}=\tau_{x}\left\{E_{L}/\tau_{L}+aK\left[x_{E}r_{E}E_{E}+x_{I}r_{I}g\gamma E_{I}\right]\right\},\;\sigma^{2}(V)\to \sigma_{x}^{2}(V)=a^{2}K\tau_{x}[y_{E}r_{E}{\left(V-E_{E}\right)}^{2}+g^{2}\gamma y_{I}r_{I}{\left(V-E_{I}\right)}^{2}],\;x_{E,I}=\frac{1}{1+Ur_{E,I}\tau_{D}},\;y_{E,I}=\frac{x_{E,I}}{1+U(1-U/2)r_{E,I}\tau_{D}}.$$

Equation (H4) shows that short-term depression affects the single-neuron response for rates *r*_*E,I*_ ~ 1/*τ*_*D*_*U* or larger. In particular, the effective time constant *τ*_*x*_ decreases monotonically with input firing rate and, for *ν*_*X*_ ≫ 1/*τ*_*D*_*U*, plateaus at

(H5)
$$\tau_{x}^{*}=\frac{U\tau_{D}}{aK(1+g\gamma )}.$$

In the main text, we show that the ratio *τ*/*τ*_*S*_ determines neural response: Activity increases (decreases) with inputs for *τ*/*τ*_*S*_ ≫ 1 (*τ*/*τ*_*S*_ ≪ 1). In the absence of short-term depression, *τ* ~ 1/*aKν*_*X*_ and the regime *τ*/*τ*_*S*_ ≪ 1 is always reached for large inputs, regardless of the value of *τ*_*S*_. Equation (H5) shows that, with short-term depression and for certain parameters, the regime *τ*/*τ*_*S*_ ≪ 1 is not reached; this suggests that short-term depression might prevent suppression of single-neuron response for large inputs. To validate this intuition, we compute numerically the response of conductance-based synapses neurons with a finite synaptic time constant and short-term depression; results are shown in Fig. 17. Simulations show that, for parameters in which short-term depression prevents the regime *τ*/*τ*_*S*_ ≪ 1 to appear, the single-neuron response is still suppressed for large *ν*_*X*_. This numerical result can be understood by noticing that, with short-term depression and for *r*_*E,I*_ ≫ 1/*τ*_*D*_*U*, the equilibrium value of the membrane potential *μ*_*x*_ remains constant, while the variance of the synaptic input $\sigma_{x}^{2}$ decreases with presynaptic input as 1/(*r*_*X*_*τ*_*D*_)^2^ [Eq. (H4)]. For parameters such that $\tau_{x}^{*}/\tau_{S}\gg 1$, these properties lead to an exponential suppression of the single-neuron firing [Eq. (12)] when inputs are large.

Results described in this section show that short-term plasticity suppresses neural response for large inputs. This suppression, unlike that generated by a finite synaptic time constant, emerges because synaptic current fluctuations become small, while the effective time constant remains finite. It follows that, in models with short-term plasticity, the autocorrelation of the membrane potential can be of the order of *τ** for large inputs and can be larger than the synaptic time constant. Finally, we point out that, analogously to models without short-term plasticity, response suppression does not appear in networks of neurons, as it is prevented by recurrent interactions.
